# Luxembourg Parkinson’s study -comprehensive baseline analysis of Parkinson’s disease and atypical parkinsonism

**DOI:** 10.3389/fneur.2023.1330321

**Published:** 2023-12-19

**Authors:** Lukas Pavelka, Rajesh Rawal, Soumyabrata Ghosh, Claire Pauly, Laure Pauly, Anne-Marie Hanff, Pierre Luc Kolber, Sonja R. Jónsdóttir, Deborah Mcintyre, Kheira Azaiz, Elodie Thiry, Liliana Vilasboas, Ekaterina Soboleva, Marijus Giraitis, Olena Tsurkalenko, Stefano Sapienza, Nico Diederich, Jochen Klucken, Enrico Glaab, Gloria A. Aguayo, Eduardo Rosales Jubal, Magali Perquin, Michel Vaillant, Patrick May, Manon Gantenbein, Venkata P. Satagopam, Rejko Krüger, on behalf of the NCER-PD Consortium

**Affiliations:** ^1^Transversal Translational Medicine, Luxembourg Institute of Health (LIH), Strassen, Luxembourg; ^2^Parkinson Research Clinic, Centre Hospitalier de Luxembourg (CHL), Strassen, Luxembourg; ^3^Translational Neuroscience, Luxembourg Centre for Systems Biomedicine (LCSB), University of Luxembourg, Esch-sur-Alzette, Luxembourg; ^4^Bioinformatics Core, Luxembourg Centre for Systems Biomedicine (LCSB), University of Luxembourg, Esch-sur-Alzette, Luxembourg; ^5^Faculty of Science, Technology and Medicine, University of Luxembourg, Esch-sur-Alzette, Luxembourg; ^6^Department of Epidemiology, CAPHRI School for Public Health and Primary Care, Maastricht University Medical Centre, Maastricht, Netherlands; ^7^Department of Neurosciences, Centre Hospitalier de Luxembourg (CHL), Strassen, Luxembourg; ^8^Department of Precision Health, Luxembourg Institute of Health (LIH), Strassen, Luxembourg; ^9^Biomedical Data Science Group, Luxembourg Centre for Systems Biomedicine (LCSB), University of Luxembourg, Esch-sur-Alzette, Luxembourg; ^10^Deep Digital Phenotyping Research Unit, Luxembourg Institute of Health (LIH), Strassen, Luxembourg; ^11^Translational Medicine Operations Hub, Luxembourg Institute of Health (LIH), Strassen, Luxembourg

**Keywords:** Parkinson’s disease, parkinsonian disorders, progressive supranuclear palsy, environment exposure, hyposmia, REM-sleep behaviour disorder

## Abstract

**Background:**

Deep phenotyping of Parkinson’s disease (PD) is essential to investigate this fastest-growing neurodegenerative disorder. Since 2015, over 800 individuals with PD and atypical parkinsonism along with more than 800 control subjects have been recruited in the frame of the observational, monocentric, nation-wide, longitudinal-prospective Luxembourg Parkinson’s study.

**Objective:**

To profile the baseline dataset and to explore risk factors, comorbidities and clinical profiles associated with PD, atypical parkinsonism and controls.

**Methods:**

Epidemiological and clinical characteristics of all 1,648 participants divided in disease and control groups were investigated. Then, a cross-sectional group comparison was performed between the three largest groups: PD, progressive supranuclear palsy (PSP) and controls. Subsequently, multiple linear and logistic regression models were fitted adjusting for confounders.

**Results:**

The mean (SD) age at onset (AAO) of PD was 62.3 (11.8) years with 15% early onset (AAO < 50 years), mean disease duration 4.90 (5.16) years, male sex 66.5% and mean MDS-UPDRS III 35.2 (16.3). For PSP, the respective values were: 67.6 (8.2) years, all PSP with AAO > 50 years, 2.80 (2.62) years, 62.7% and 53.3 (19.5). The highest frequency of hyposmia was detected in PD followed by PSP and controls (72.9%; 53.2%; 14.7%), challenging the use of hyposmia as discriminating feature in PD vs. PSP. Alcohol abstinence was significantly higher in PD than controls (17.6 vs. 12.9%, *p* = 0.003).

**Conclusion:**

Luxembourg Parkinson’s study constitutes a valuable resource to strengthen the understanding of complex traits in the aforementioned neurodegenerative disorders. It corroborated several previously observed clinical profiles, and provided insight on frequency of hyposmia in PSP and dietary habits, such as alcohol abstinence in PD.

**Clinical trial registration**: clinicaltrials.gov, NCT05266872.

## Introduction

The global incidence and prevalence of Parkinson’s disease (PD) has been likened to a pandemic, with the number of affected individuals rising from 2.6 million in 1990 to 6.5 million in 2016. This number is projected to surge to 17 million by 2040 (1). Due to the chronic and progressive nature of the disease, which significantly afflicts patients, their families, and society, it is crucial to prioritize the understanding of its aetiology. About 20 to 30% of all PD cases stem from the genetic mutations, combining both monogenic and polygenic causes (2, 3), leaving 70% classified as ‘idiopathic’. To develop targeted symptomatic and disease-modifying treatments, in-depth phenotyping, using multiscale clinical and biological data from extensive longitudinal PD cohorts is essential. This approach fosters the implementation of the precision medicine concept, within the field of neurodegenerative disorders (4).

Beyond the genetic background, several risk factors have been strongly linked to PD including traumatic brain injury (5), exposure to pesticides (6, 7) and heavy metals (8). Among potential protective factors, smoking (9–11), caffeine intake (12–14) and physical activity (15) are often listed. However, the relationship between PD and comorbidities such as diabetes mellitus, cardiovascular disease and cancers (excluding melanoma) remains controversial, thus requiring further epidemiological studies (16). To this end, the Luxembourg Parkinson’s study was established in 2015 in the framework of the National Centre of Excellence in Research in Parkinson’s disease (NCER-PD). The Luxembourg Parkinson’s study is a monocentric, observational cohort of individuals with neurodegenerative parkinsonism (PS) and controls without manifested clinical evidence for a neurodegenerative disorder (17). The primary objective of the Luxembourg Parkinson’s study has been an in-depth phenotyping of people with PS in order to have a better understanding of the neurodegenerative process at multiple clinical and biological levels.

Whereas general cohort setup, methods and recruitment was published by our group in 2018 (17), this study aimed to (i) present a general overview of the Luxembourg Parkinson’s study baseline dataset and (ii) to perform an epidemiological association analysis assessing the socio-demographic characteristics, environmental exposures, comorbidities, and clinical profiles of the three major diagnostic groups in this dataset: PD, progressive supranuclear palsy (PSP), and controls.

## Materials and methods

### Recruitment and ethical considerations

The participants were sequentially enrolled in the study baseline dataset until March 2021. Participants were recruited from Luxembourg and the surrounding geographical areas of Germany, France, and Belgium, as defined by the Greater Region. All study subjects signed a written informed consent. The study was approved by the National Ethics Board in Luxembourg (CNER Ref: 201407/13) and complied with Declaration of Helsinki. Luxembourg Parkinson’s study was registered in ClinicalTrials.gov under NCT05266872.

### Group definitions, inclusion, and exclusion criteria

All patients enrolled in the study underwent diagnostic evaluation and were assigned a clinical diagnosis based on established criteria, as follows: PD was based on UK Parkinson’s Disease Society Brain Bank (UKPDSBB) (18); for PSP, Institute of Neurological Disorders and Stroke Society criteria (19) with additional classification of the basal syndrome (CBS) as PSP, based on Movement Disorder Society (MDS) criteria for PSP in 2017 (20); for frontotemporal dementia with PS [FTD-P; (21)]; for multiple system atrophy [MSA; (22)]; for dementia with Lewy bodies [DLB; (23)]; for vascular PS [VaP; (24)] and for initial diagnostic and follow-up evaluation of drug-induced PS (25). Controls were defined as individuals >18 years old with no evidence of a neurodegenerative disorder and no active cancer at the time of inclusion.

Secondary PS was excluded, i.e., drug-induced or PS due to a space-occupying lesion. Individuals diagnosed with VaP were followed up, given the fact that the burden of microvascular white matter lesions together with lower-body PS are not definite markers of non-degenerative PS, as shown in several autopsy studies (26). *De novo* PD were defined as dopaminergic-medication naïve patients within 1 year since diagnosis. Detailed recruitment strategy as well as inclusion and exclusion criteria in Luxembourg Parkinson’s study were previously described in Hipp et al. (17).

### Clinical investigations and definition of variables

Information on socio-demographics, comorbidities and clinical profile of PS with assessment of motor and non-motor symptoms at the time or before the on-site diagnostic evaluation were acquired during a semi-structured interview and neurological examination by a study physician. Neuropsychological examination including the Montreal Cognitive Assessment (MoCA) as well as olfactory function were assessed by neuropsychologists, study physicians and research nurses specialized in PD. Olfactory function was examined with 16 items Sniffin’ Stick Identification test, defining hyposmia as below the 10^th^ percentile with age-related cut-offs: for age group ≤ 35 years (cut-off ≤ 11); > 35 years and < 55 (cut-off ≤ 12); for age group ≥ 55 (cut-off ≤ 9) (27). Age at disease onset (AAO) was defined as age at diagnosis of the neurodegenerative disorder. Probable Rapid Eye Movement (REM)-Sleep Behaviour Disorder (pRBD) was defined based on the RBD Screening Questionnaire total score (RBDSQ) ≥ 6 for patient groups and RBDSQ ≥ 5 for controls (28). The information on environmental exposure and medication use was based on the modified PD Risk Factor Questionnaire (PD-RFQ-U) Epi Info™ developed by Caroline Tanner (17). Life-long alcohol abstinence was defined as intake of fewer than 100 alcoholic beverages over a lifetime. Regular intake of alcohol was defined as at least one drink per week for 6 months or longer. Regular intake of non-dopaminergic medication was defined as intake of at least two pills per week for 6 months or longer. History of exposure to pesticides corresponded to the reported at-home or occupational use of any type of pesticides, insecticides, fungicides, herbicides, rodenticides or fumigants. Exposure to (i) solvents or degreasers; (ii) welded, brazed or flame cut metal; (iii) regular solder activity; (iv) metal dust or metal fumes; (v) exposure to metals not otherwise specified were defined as exposure at least for 100 or more days at work and/or at home. Definition of obesity was based on Body Mass Index (BMI) ≥ 30. Metabolic syndrome was defined as positive for all three following comorbidities: diabetes, arterial hypertension and obesity based on BMI. A further detailed description of the clinical symptoms and assessment scales are provided in the Supplementary material. All on-site assessments were conducted in medication ON-state and, where applicable, deep brain stimulation ON-state. The calculation of levodopa equivalent daily dose (LEDD, reported in gram/day where not stated otherwise) was based on previously published conversion factors (29). The patient or a delegated person completed standardized self-reporting questionnaires assessing quality of life, activities of daily living, motor and non-motor symptoms and environmental exposures (see Supplementary material for further details).

### Data monitoring process

All clinical data were captured and encoded in pseudonymized form in the secured online platform using REDCap electronic data capture tools hosted at University of Luxembourg (30, 31). Furthermore, the pseudonymized data underwent two-step monitoring process for data completeness and accuracy as described in detail in the Supplementary material.

### Missing data handling

**Figure 1:**
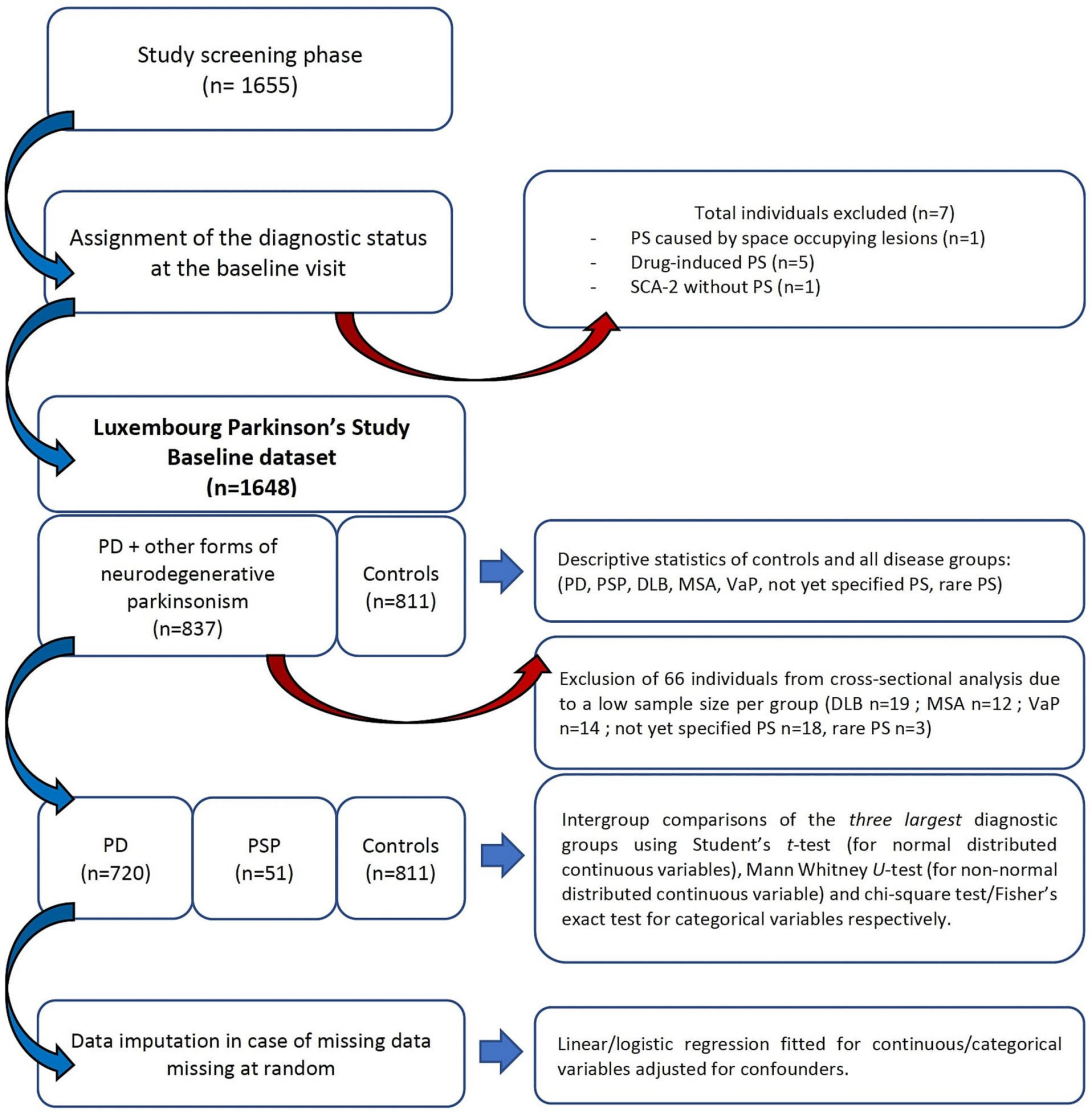
Recruitment flowchart and analytical steps of the Luxembourg Parkinson’s study baseline dataset. Parkinson’s disease (PD); progressive supranuclear palsy (PSP); parkinsonism (PS), vascular parkinsonism (VaP); dementia with Lewy Bodies (DLB); multiple system atrophy (MSA); spinocerebellar ataxia (SCA).

The number and percentage of missing data per variable (Supplementary Table S2) and their association with relevant clinical outcomes (Hoehn & Yahr, MDS-UPDRS III and MoCA) were described for each variable with missing data >5% (Supplementary Table S3). In the cross-sectional analysis of PD, PSP and controls, missing at-random mechanism was assumed as the missing data can be inferred from the information present in our dataset. Previous research in neurocognitive diseases demonstrated that ignoring missing data, when the missing values were correlated to the outcome, can lead to bias (32). Therefore, multiple imputations of missing data were considered as the best way to address the bias via multivariate Imputation by Chained Equations (MICE) R package (33) with imputation using 10 iterations and 5 imputed datasets. Values were not imputed when the test was not planned due to impossibility of test performance (e.g., amputated hand when assessing MDS-UPDRS III or vision problem/blindness in cognitive assessment; annotated as not-testable). The individuals with not-testable values were excluded from the analysis before fitting the regression analysis (see in the analysis workflow in Figure 1).

### Statistical analysis

The *compareGroups* R package was used for the univariate analyses (34). Diagnostic groups MSA, DLB, VaP, and rare PS were exempted from the comparative statistics due to a low sample size per group. For unpaired two group comparisons (PD vs. controls, PD vs. PSP and PSP vs. controls), odds ratios (OR), confidence intervals at 95% (CI) and *p*-values were obtained for each variable. Prediction model pooling of linear and logistic regression was used for continuous and categorical variables (“psfmi”) R package (35). Likelihood ratio statistics were pooled with the Meng and Rubin method and the median *p*-values were pooled using the Median P Rule (MPR) (36, 37). We accounted for multiple comparisons using the Bonferroni correction. The regression analyses on all outcomes were adjusted for sex, age at assessment (AAA) and total languages spoken (TLS) in regression models using PD vs. controls and PSP vs. controls. By contrast, PD vs. PSP was adjusted for sex, AAA, TLS and disease duration.

## Results

**Figure 2:**
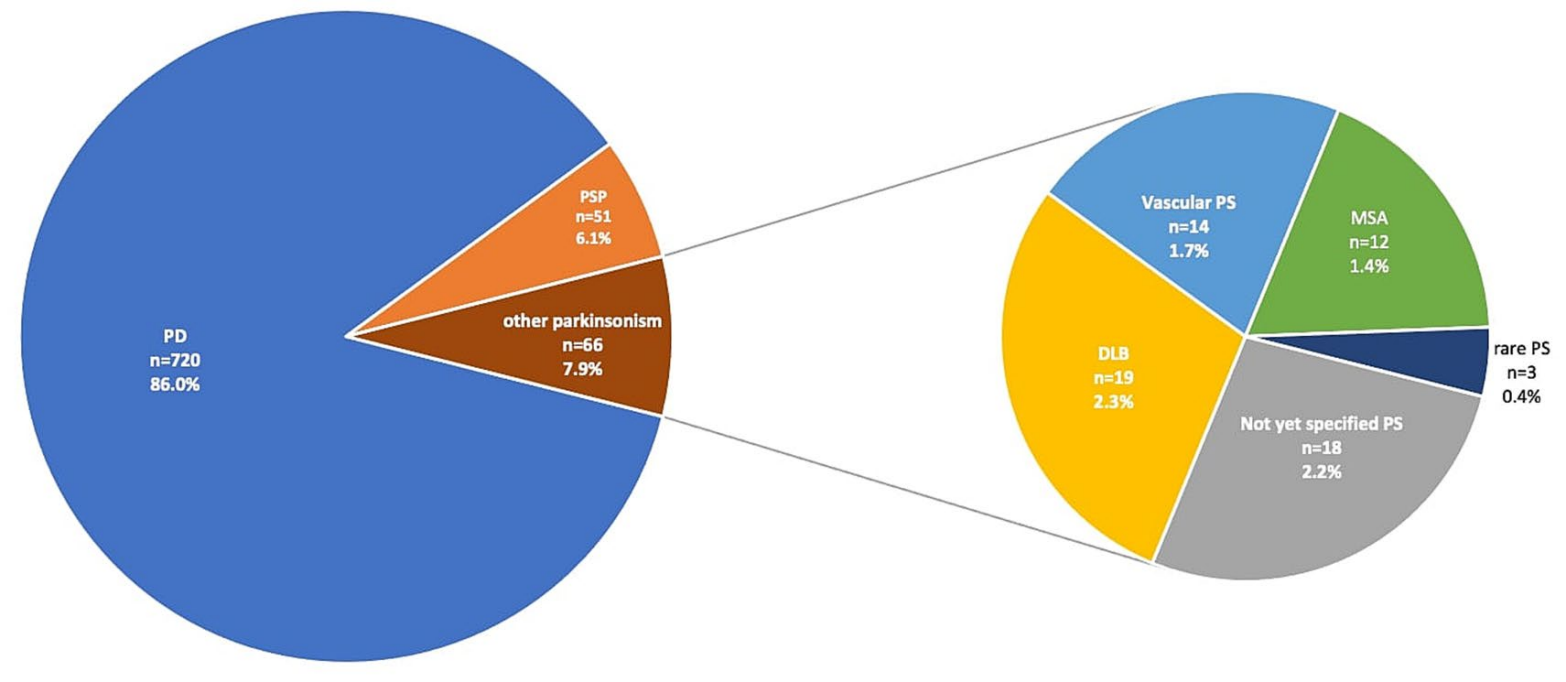
Distribution of diagnostic groups with parkinsonism (PS) in the baseline dataset of Luxembourg Parkinson’s study. The annotations correspond to diagnostic group, number of individuals (*n*) and the proportion (%) to the overall individuals with PS in the baseline dataset. Parkinson’s disease (PD); progressive supranuclear palsy (PSP; including corticobasal syndrome under PSP based on MDS diagnostic criteria from 2017); dementia with Lewy Bodies (DLB); multiple system atrophy (MSA); rare PS includes one case of rapid-onset dystonia-parkinsonism with DYT12 mutation, one case of chronic progressive external ophthalmoplegia (CPEO) and one case of frontotemporal dementia with PS.

We enrolled 1,655 participants in the study screening phase of the Luxembourg Parkinson’s study. After diagnostic evaluation at the baseline visit and application of exclusion criteria, 837 patients with PS and 811 controls were included in the baseline dataset. The PS group comprised 86% individuals with PD, 6.1% with PSP, 2.3% DLB, 1.4% MSA, 1.7% VaP, 0.4% rare PS [cases of rapid onset dystonia-PS (DYT12, *n* = 1), chronic progressive external ophthalmoplegia (*n* = 1) and frontotemporal dementia with PS (*n* = 1)] and 2.2% not yet specified cases with PS (Figure 2). The socio-demographic data, comorbidities, clinical characteristics and environmental exposure of the three largest groups, i.e., PD, controls, and PSP (43.7, 49.2 and 3.1% of the baseline dataset, respectively) are shown in Tables 1–4. The descriptive characteristics of MSA, DLB, VaP, and rare PS were included in Supplementary Tables S4–S6.

**Table 1:** Sociodemographic characteristics and environmental exposure in Luxembourg Parkinson’s study baseline dataset.

	PD mean (SD) or YES/NO (%)	PSP mean (SD) or YES/NO (%)	Controls mean (SD) or YES/NO (%)	PD vs. control OR [95% CI]	PSP vs. control OR [95% CI]	PD vs. PSP OR [95% CI]
Individuals per group	*N* = 720	*N* = 51	*N* = 811	-	-	-
Sex (male)	479/241 (66.5%)	32/19 (62.7%)	423/388 (52.2%)	1.82 [1.48;2.24] *	1.54 [0.86;2.82]	1.19 [0.61;2.24]
Age at assessment (years)	67.3 (10.9)	70.4 (7.54)	59.7 (12.1)	1.06 [1.05;1.07] *	1.11 [1.07;1.15] *	0.97 [0.94;1.00]
Family history of parkinsonism	192/527 (26.7%)	9/40 (18.4%)	276/533 (34.1%)	0.70 [0.56;0.88]	0.44 [0.20;0.88]	1.60 [0.79;3.59]
Family history of dementia	189/524 (26.5%)	12/37 (24.5%)	275/530 (34.2%)	0.70 [0.56;0.87]	0.63 [0.31;1.20]	1.10 [0.58;2.26]
Years of education completed	12.9 (4.08)	11.6 (3.98)	14.3 (3.84)	0.91 [0.89;0.94] *	0.82 [0.76;0.89] *	1.08 [1.01;1.16]
Total languages spoken	2.83 (1.06)	2.78 (0.86)	3.50 (0.81)	0.47 [0.42;0.53] *	0.47 [0.36;0.61] *	1.04 [0.79;1.36]
Regular intake of caffeinated beverages	631/55 (92.0%)	46/2 (95.8%)	768/34 (95.8%)	0.51 [0.32;0.79]	0.95 [0.28;6.47]	0.53 [0.08;1.80]
History or presence of smoking	325/358 (47.6%)	21/27 (43.8%)	383/419 (47.8%)	0.99 [0.81;1.22]	0.85 [0.47;1.53]	1.17 [0.65;2.13]
Life-long alcohol abstinence	117/547 (17.6%)	14/31 (31.1%)	101/682 (12.9%)	1.44 [1.08;1.93]	3.06 [1.52;5.87]	0.47 [0.25;0.94]
Regular intake of alcohol	435/238 (64.6%)	31/16 (66.0%)	541/248 (68.6%)	0.84 [0.67;1.04]	0.88 [0.48;1.69]	0.95 [0.49;1.75]
Regular intake of aspirin	122/537 (18.2%)	8/36 (18.2%)	83/711 (10.5%)	1.91 [1.41;2.58] *	1.93 [0.80;4.11]	0.99 [0.47;2.35]
Regular intake of ibuprofen-based non-aspirin medications	89/567 (13.6%)	7/36 (16.3%)	89/692 (11.4%)	1.22 [0.89;1.67]	1.54 [0.61;3.38]	0.79 [0.36;2.02]
Regular intake of other anti-inflammatory medication	112/547 (17.0%)	9/34 (20.9%)	83/701 (10.6%)	1.73 [1.27;2.35] *	2.26 [0.98;4.71]	0.76 [0.37;1.75]
History of calcium channel blocker intake	315/262 (47.2%)	22/22 (50.0%)	320/471 (40.5%)	1.32 [1.07;1.62]	1.47 [0.80;2.72]	0.90 [0.48;1.66]
History of exposure to pesticides	417/269 (60.8%)	28/20 (58.3%)	547/255 (68.2%)	0.72 [0.58;0.89]	0.65 [0.36;1.20]	1.11 [0.60;2.01]
Exposure to glues or adhesives	68/595 (10.3%)	3/42 (6.67%)	61/725 (7.76%)	1.36 [0.94;1.96]	0.89 [0.20;2.55]	1.53 [0.54;6.67]
Exposure to solvents or degreasers	98/562 (14.8%)	7/38 (15.6%)	94/687 (12.0%)	1.27 [0.94;1.73]	1.37 [0.54;2.99]	0.93 [0.43;2.35]
Exposure to welded, brazed or flame cut metal	71/606 (10.5%)	6/41 (12.8%)	45/753 (5.64%)	1.96 [1.33;2.91] *	2.49 [0.90;5.82]	0.78 [0.34;2.14]
Exposure to regular solder activity	53/625 (7.82%)	1/44 (2.22%)	32/767 (4.01%)	2.03 [1.30;3.22]	0.62 [0.03;2.97]	3.27 [0.70;77.4]
Exposure to metal dust or metal fumes	116/549 (17.4%)	13/34 (27.7%)	85/708 (10.7%)	1.76 [1.30;2.38] *	3.20 [1.57;6.19]	0.55 [0.29;1.11]
Exposure to metal not otherwise categorized	137/509 (21.2%)	9/36 (20.0%)	128/644 (16.6%)	1.35 [1.04;1.77]	1.27 [0.56;2.61]	1.06 [0.52;2.42]

Intergroup comparisons using Student’s t-test (for normal distributed continuous variables), Mann Whitney U-test (for non-normal distributed continuous variables) and chi-square test or Fisher’s exact test for categorical variables, respectively. Statistically significant is indicated by an asterisk after Bonferroni correction for multiple testing. PD (Parkinson’s disease); PSP (progressive supranuclear palsy); OR (odds ratio); CI (95% confidence interval). Variables were defined in Methods or in Supplementary material.

**Table 2:** Clinical characteristics, motor and non-motor symptoms/scales in Luxembourg Parkinson’s study baseline dataset.

	PD mean (SD) or YES/NO (%)	PSP mean (SD) or YES/NO (%)	Controls Mean (SD) or YES/NO (%)	PD vs. controls OR [95% CI]	PSP vs. controls OR [95% CI]	PD vs. PSP OR [95% CI]
Individuals per group	*n* = 720	*n* = 51	*n* = 811	-	-	-
Age at onset (years)	62.3 (11.8)	67.6 (8.20)	-	-	-	0.96 [0.93;0.98] *
Hoehn & Yahr stage (H&Y)	2.21 (0.79)	3.30 (1.26)	-	-	-	0.36 [0.28;0.47] *
Disease duration since diagnosis (years)	4.90 (5.16)	2.80 (2.62)	-	-	-	1.13 [1.04;1.23] *
MDS-UPDRS I	10.6 (7.02)	16.5 (8.60)	37 (2.245)	1.20 [1.18;1.23] *	1.27 [1.21;1.33] *	0.91 [0.88;0.95] *
MDS-UPDRS II	11.5 (8.36)	23.7 (12.3)	29 (1.76)	1.63 [1.55;1.71] *	1.45 [1.34;1.57] *	0.90 [0.87;0.92] *
MDS-UPDRS III	35.2 (16.3)	53.3 (19.5)	38 (2.306)	1.36 [1.32;1.41] *	1.25 [1.18;1.32] *	0.95 [0.93;0.96] *
MDS-UPDRS IV	1.68 (3.29)	2.02 (3.86)	22 (1.335)	-	-	0.97 [0.90;1.05]
*De novo* PD patient	73/647 (10.1%)	-	-	-	-	-
LEDD gram/day	0.50 (0.41)	0.46 (0.39)	0.00 (0.04)	-	-	1.29 [0.62;2.68]
Deep Brain Stimulation (DBS)	29/691 (4.03%)	-	-	-	-	-
Treatment with pump^1^	4/716 (0.55%)	-	-	-	-	-
Metabolic syndrome	28/692 (3.89%)	2/48 (4.00%)	25/785 (3.09%)	1.27 [0.73;2.22]	1.40 [0.20;4.91]	0.91 [0.26;6.21]
BMI (kg/m^2^)	27.6 (4.74)	27.2 (4.17)	27.3 (5.15)	1.02 [0.99;1.04]	1.00 [0.94;1.06]	1.02 [0.96;1.09]
Obesity	184/514 (26.4%)	11/34 (24.4%)	201/608 (24.8%)	1.08 [0.86;1.37]	0.99 [0.47;1.93]	1.10 [0.56;2.32]
MoCA	24.3 (4.55)	20.0 (6.29)	27.1 (2.53)	0.78 [0.76;0.81] *	0.65 [0.59;0.71] *	1.18 [1.12;1.25] *
Sniffin’ Stick test	7.87 (3.52)	9.60 (3.56)	12.7 (2.37)	0.59 [0.56;0.62] *	0.74 [0.68;0.81] *	0.87 [0.79;0.95] *
Hyposmia	512/190 (72.9%)	25/22 (53.2%)	119/689 (14.7%)	15.6 [12.1;20.2] *	6.55 [3.57;12.1] *	2.37 [1.29;4.32]
NMSS	9.65 (5.53)	11.0 (4.31)	4.57 (4.14)	1.24 [1.21;1.27] *	1.27 [1.19;1.35] *	0.96 [0.90;1.01]
RBDSQ	4.55 (3.16)	3.84 (2.74)	2.63 (2.36)	1.29 [1.23;1.34] *	1.19 [1.07;1.32]	1.08 [0.97;1.21]
PDSS	105 (24.8)	98.0 (27.9)	122 (18.9)	0.96 [0.96;0.97] *	0.96 [0.95;0.97] *	1.01 [1.00;1.02]
BDI-I	9.89 (7.06)	16.3 (8.20)	5.52 (5.41)	1.13 [1.10;1.15]*	1.20 [1.15;1.25] *	0.91 [0.88;0.94] *
SCOPA-AUT	14.9 (8.38)	18.8 (8.82)	7.82 (6.12)	1.15 [1.13;1.17] *	1.16 [1.12;1.21] *	0.95 [0.92;0.99]
PDQ-39	39.3 (26.5)	67.8 (28.4)	11.0 (13.6)	1.08 [1.07;1.09] *	1.09 [1.07;1.12] *	0.97 [0.96;0.98] *
Starkstein Apathy scale	14.1 (5.86)	18.3 (6.61)	9.65 (4.65)	1.18 [1.16;1.21] *	1.32 [1.24;1.41] *	0.90 [0.86;0.95] *

Intergroup comparisons using Student’s t-test (for normal distributed continuous variables), Mann Whitney U-test (for non-normal distributed continuous variables) and chi-square test or Fisher’s exact test for categorical variables, respectively. Statistically significant is indicated by an asterisk after Bonferroni correction for multiple testing. ^1^two PD patients with apomorphine pump and two PD with continuous levodopa/carbidopa jejunal pump. PD (Parkinson’s disease); PSP (progressive supranuclear palsy); OR (odds ratio); CI (95% confidence interval). Symptoms and scales were defined in Methods or in Supplementary material.

**Table 3:** Current motor symptoms and non-motor symptoms of patients with Parkinson’s disease (PD), progressive supranuclear palsy (PSP) and controls in Luxembourg Parkinson’s study baseline dataset.

	PD Mean (SD) or YES/NO (%)	PSP Mean (SD) or YES/NO (%)	Controls Mean (SD) or YES/NO (%)	PD vs. controls OR [95% CI]	PSP vs. controls OR [95% CI]	PD vs. PSP OR [95% CI]
Individuals per group	*n* = 720	*n* = 51	*n* = 811	-	-	-
Gait disorder	401/318 (55.8%)	40/11 (78.4%)	22/789 (2.71%)	44.8 [29.3;72.2] *	126 [59.0;290] *	0.35 [0.17;0.67]
Freezing of Gait	164/555 (22.8%)	15/36 (29.4%)	0/811 (0.00%)	-	-	0.71 [0.38;1.36]
Repetitive falls	127/592 (17.7%)	35/16 (68.6%)	8/803 (0.99%)	21.1 [10.9;47.6] *	209 [87.7;561] *	0.10 [0.05;0.18] *
Dyskinesias	87/632 (12.1%)	2/49 (3.92%)	0/811 (0.00%)	-	-	3.14 [0.95;20.9]
Motor Fluctuations	120/599 (16.7%)	4/47 (7.84%)	0/811 (0.00%)	-	-	2.27 [0.90;7.80]
Dysphagia	184/535 (25.6%)	26/25 (51.0%)	12/799 (1.48%)	22.6 [13.0;43.3] *	67.4 [31.1;155] *	0.33 [0.19;0.59] *
probable RBD	219/437 (33.4%)	10/33 (23.3%)	135/646 (17.3%)	2.40 [1.87;3.07] *	1.46 [0.67;2.95]	1.64 [0.82;3.58]
Excessive daily sleepiness	229/490 (31.8%)	15/36 (29.4%)	22/789 (2.71%)	16.6 [10.8;26.9] *	14.8 [6.98;31.1] *	1.11 [0.61;2.15]
Insomnia	194/525 (27.0%)	11/40 (21.6%)	72/739 (8.88%)	3.78 [2.84;5.10] *	2.84 [1.33;5.63]	1.33 [0.69;2.79]
Orthostatic hypotension	212/ (29.5%)	11/40 (21.6%)	53/758 (6.54%)	5.96 [4.35;8.29] *	3.96 [1.83;7.96] *	1.50 [0.78;3.15]
Syncope	36/683 (5.01%)	3/48 (5.88%)	9/802 (1.11%)	4.63 [2.31;10.4] *	5.73 [1.18;20.3]	0.81 [0.28;3.57]
Constipation	308/411 (42.8%)	26/25 (51.0%)	53/758 (6.54%)	10.7 [7.85;14.8] *	14.8 [7.95;27.6] *	0.72 [0.41;1.28]
Urinary incontinence	230/489 (32.0%)	22/29 (43.1%)	39/772 (4.81%)	9.27 [6.55;13.4] *	14.9 [7.79;28.4] *	0.62 [0.35;1.12]
Hallucinations	116/603 (16.1%)	5/46 (9.80%)	3/808 (0.37%)	49.2 [18.5;206] *	28.4 [6.49;150] *	1.72 [0.73;5.12]
Impulse control disorder	67/652 (9.32%)	2/49 (3.92%)	2/809 (0.25%)	38.6 [12.1;253] *	16.4 [1.67;161]	2.35 [0.70;15.7]

Intergroup comparisons using Student’s t-test (for normal distributed continuous variables), Mann Whitney U-test (for non-normal distributed continuous variables) and chi-square test or Fisher’s exact test for categorical variables, respectively. Statistically significant is indicated by an asterisk after Bonferroni correction for multiple testing. PD (Parkinson’s disease); PSP (progressive supranuclear palsy); OR (odds ratio); CI (95% confidence interval). Symptoms were defined in Methods or Supplementary material.

**Table 4:** Overview of comorbidities in patients with Parkinson’s disease (PD), progressive supranuclear palsy (PSP) and controls.

	PD Mean (SD) or YES/NO (%)	PSP Mean (SD) or YES/NO (%)	Controls Mean (SD) or YES/NO (%)	PD vs. controls OR [CI]	PSP vs. controls OR [CI]	PD vs. PSP OR [CI]
Individuals per group	*n* = 720	*n* = 51	*n* = 811	-	-	-
History of restless legs syndrome	63/656 (8.76%)	5/46 (9.80%)	29/782 (3.58%)	2.58 [1.66;4.11] *	3.00 [0.97;7.54]	0.86 [0.36;2.59]
Diabetes	73/646 (10.2%)	8/43 (15.7%)	53/758 (6.54%)	1.61 [1.12;2.35]	2.69 [1.12;5.77]	0.60 [0.28;1.43]
Arterial Hypertension	307/412 (42.7%)	27/24 (52.9%)	249/562 (30.7%)	1.68 [1.36;2.08] *	2.53 [1.43;4.52]	0.66 [0.37;1.18]
Cardiovascular disease	142/577 (19.7%)	10/41 (19.6%)	79/732 (9.74%)	2.28 [1.70;3.07] *	2.28 [1.04;4.58]	1.00 [0.51;2.16]
Hypercholesterolemia	281/438 (39.1%)	17/34 (33.3%)	299/512 (36.9%)	1.10 [0.89;1.35]	0.86 [0.46;1.55]	1.28 [0.71;2.39]
Epileptic seizures	17/702 (2.36%)	0/51 (0.00%)	16/795 (1.97%)	1.20 [0.60;2.43]	-	-
History of stroke	31/688 (4.31%)	6/45 (11.8%)	23/788 (2.84%)	1.54 [0.89;2.70]	4.63 [1.62;11.4]	0.33 [0.14;0.93]
Traumatic brain injury	167/552 (23.2%)	12/39 (23.5%)	173/638 (21.3%)	1.12 [0.88;1.42]	1.14 [0.56;2.18]	0.97 [0.51;1.99]
History of cancer^1^	91/628 (12.7%)	6/45 (11.8%)	65/746 (8.01%)	1.66 [1.19;2.33]	1.56 [0.57;3.56]	1.06 [0.47;2.88]
History of melanoma	8/711 (1.11%)	1/50 (1.96%)	14/797 (1.73%)	0.65 [0.25;1.53]	1.29 [0.05;6.64]	0.50 [0.09;12.8]
History of prostate cancer	34/685 (4.73%)	3/48 (5.88%)	14/797 (1.73%)	2.81 [1.52;5.46]	3.69 [0.79;11.9]	0.76 [0.26;3.37]
History of brain cancer	12/707 (1.67%)	1/50 (1.96%)	10/801 (1.23%)	1.36 [0.58;3.26]	1.80 [0.07;9.83]	0.75 [0.14;18.7]
History of breast cancer	17/702 (2.36%)	1/50 (1.96%)	11/800 (1.36%)	1.75 [0.82;3.91]	1.64 [0.07;8.78]	1.07 [0.21;26.0]
History of cancer not categorized	26/693 (3.62%)	0/51 (0.00%)	18/793 (2.22%)	1.65 [0.90;3.09]	-	-

Intergroup comparisons using Student’s t-test (for normal distributed continuous variables), Mann Whitney U-test (for non-normal distributed continuous variables) and chi-square test or Fisher’s exact test for categorical variables, respectively. Statistically significant is indicated by an asterisk after Bonferroni correction for multiple testing. ^1^History of cancer corresponds to the number of individuals having history of at least one cancer. PD (Parkinson’s disease); PSP (progressive supranuclear palsy); OR (odds ratio); CI (95% confidence interval).

### Baseline dataset of people with PD – sociodemographic and clinical characteristics

A total of 720 individuals with PD were recruited with the mean (standard deviation) AAO 62.3 (11.8) years, with 15% presenting early AAO [based on MDS Taskforce for Early Onset PD defined as AAO < 50 years (38)] and the mean disease duration since diagnosis 4.90 (5.16) years. The proportion of males vs. females in the PD dataset was 66.5 vs. 33.5%. All disease stages of PD as defined by Hoehn and Yahr (H&Y) were covered in the baseline dataset with mean H&Y 2.21 (0.79), where 82.5% were of early-stage PD (defined as H&Y < 3). *De novo* PD amounted to 10.1% of all PD patients and overall mean LEDD was calculated at 500 (410) mg/day. Ongoing advanced treatment via deep-brain stimulation (DBS) or via pumps (continuous levodopa/carbidopa pump or apomorphine pump) was relatively underrepresented (4.03 and 0.55% respectively). The three most frequent motor symptoms (excluding the rest tremor, bradykinesia and rigidity as defining features of PS) were gait disorder (55.8%), dysphagia (25.6%) and freezing of gait (22.8%). The top three non-motor symptoms included sleep disturbance, specifically pRBD (33.4%), urinary incontinence (32%) and excessive daily sleepiness (31.8%). With regard to the comorbidities, arterial hypertension, hypercholesterolemia and history of traumatic brain injury (42.7, 39.1 and 23.2% respectively) were identified among the most frequent. History of presence of restless legs syndrome (RLS) was high with frequency of 8.76%.

### Baseline dataset of control subjects – sociodemographic and clinical characteristics

Eight-hundred and eleven controls were included into the baseline dataset with the mean AAA 59.7 (12.1) years and male vs. female proportion 52.2 vs. 47.8%. The highest frequency of symptoms classified as non-motor symptoms for patient groups were pRBD, insomnia, orthostatic hypotension and constipation (17.3, 8.88, 6.54, and 6.54% respectively). In terms of comorbidities, the three most frequently reported were hypercholesterolemia (36.9%), arterial hypertension (30.7%) and traumatic brain injury (21.3%). RLS was identified in 3.58%. High exposure to pesticides was observed in controls vs. PD and vs. PSP (68.2 vs. 60.8 and vs. 58.3% respectively), therefore a sub-analysis was conducted to inquire into this observation as stated below.

### Intergroup comparison between PD and controls

**Figure 3:**
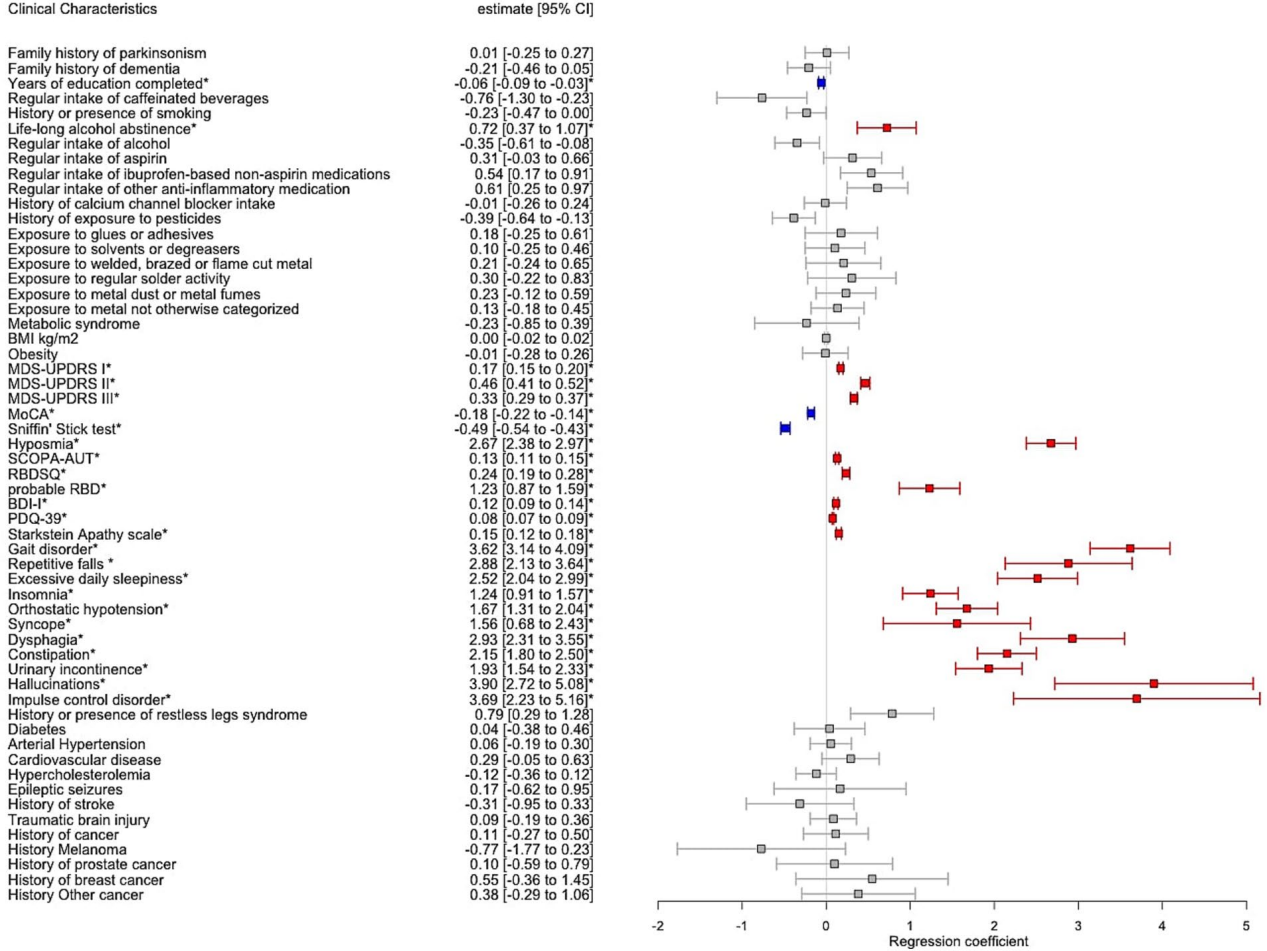
Forest plot with results of multiple regression model including Parkinson’s disease individuals (PD) vs. controls adjusted for age at assessment, sex and total languages spoken. The estimates correspond to the regression coefficient with 95% confidence intervals (95% CI). Significant associations after Bonferroni correction for multiple testing were annotated by an asterisk where red colour indicates positive significant association and blue colour negative significant association, respectively, between PD vs. controls and the clinical variable.

The recruitment of PD and controls was guided using a stratification by age categories (18–25; 26–35; 36–45; 46–55; 56–65; >65 years) and sex aiming to match the age/sex groups (Supplementary Figure S1; Supplementary Table S1). However, when comparing the mean AAA, the PD group was significantly older than the controls (mean 67.3 (10.9) vs. 59.7 (12.1) years, *p* < 0.001) and had a significantly higher proportion of males vs. females (66.5 vs. 52.2%, *p* < 0.001). For this reason, we investigated the association of PD and controls on chosen outcome variables in a multiple regression model adjusting for AAA and sex. Additionally, TLS and total years of education were significantly lower in PD vs. controls (mean 2.83 vs. 3.5, *p* < 0.001; 12.9 vs. 14.3 years, *p* < 0.001 respectively). While TLS and years of education are inherently dependent, we chose TLS as representative covariate across all regression models due to the lower OR [95% CI] in PD vs. controls (TLS: OR 0.47 [0.42–0.53]; education: OR 0.91 [0.89–0.94]). As a result, we found PD vs. controls to report higher alcohol abstinence over the lifetime (17.6 vs. 12.9%; *p* = 0.003), whereas regular alcohol intake (over the 6 last months or longer) independent of the amount was not shown to be significantly different. The remaining outcomes significantly associated with PD, reflecting the motor and non-motor symptoms and cognitive decline were shown in Figure 3.

### Sub-analysis of the pesticide exposure in PD vs. controls

Given a surprisingly high proportion of controls vs. PD reporting positive exposure to pesticides (68.2 vs. 60.8%), we conducted a sub-analysis of pesticide exposure, stratifying it by the use of pesticides at-home and in occupational settings. We identified a significantly higher occupational use of pesticides in PD compared to controls (13 vs. 8.73%, *p* = 0.04). Interestingly, the at-home use was significantly in the opposite direction, showing higher pesticides use at-home in controls compared to PD (66.6 vs. 58.9%, *p* = 0.01). But finally, none of the variables reporting on pesticide use remained significant in the regression model adjusting for AAA, sex and TLS (Supplementary Tables S7–S10; Supplementary Figures S2, S3).

### Baseline dataset of people with PSP - sociodemographic and clinical characteristics

Fifty-one individuals classified as PSP were included in the baseline dataset with clinical profile described in Tables 1–4. The mean AAO of 67.6 (8.2) years and a mean disease duration since diagnosis of 2.8 (2.6) years. The proportion of males vs. females was 62.7 vs. 37.3%. Disease severity of PSP was very high already at the time of inclusion with mean H&Y 3.3 (1.26), where 62.7% were individuals in an advanced stage of the disease (defined as H&Y ≥ 3). The overall motor impairment reflected by mean MDS-UPDRS III was 53.3 (19.5) points with a mean LEDD 460 (390) mg/day comparable to the dopaminergic treatment in the PD group (LEDD 500 (410) mg/day, *p* > 0.05). The motor symptoms with highest frequency were gait disorder (78.4%), repetitive falls (68.6%) and dysphagia (51%). Constipation (51%), urinary incontinence (43.1%) and excessive daily sleepiness (29.4%) were found to be among the three most frequent non-motor symptoms. Similar to the PD group, arterial hypertension, hypercholesterolemia and history of traumatic brain injury (52.9, 33.3, and 23.5% respectively) were determined to be among the most frequent comorbidities. From the sleep disturbances, RLS and pRBD were commonly reported in PSP (9.8 and 23.3% respectively).

### Intergroup comparison between PD and PSP

**Figure 4:**
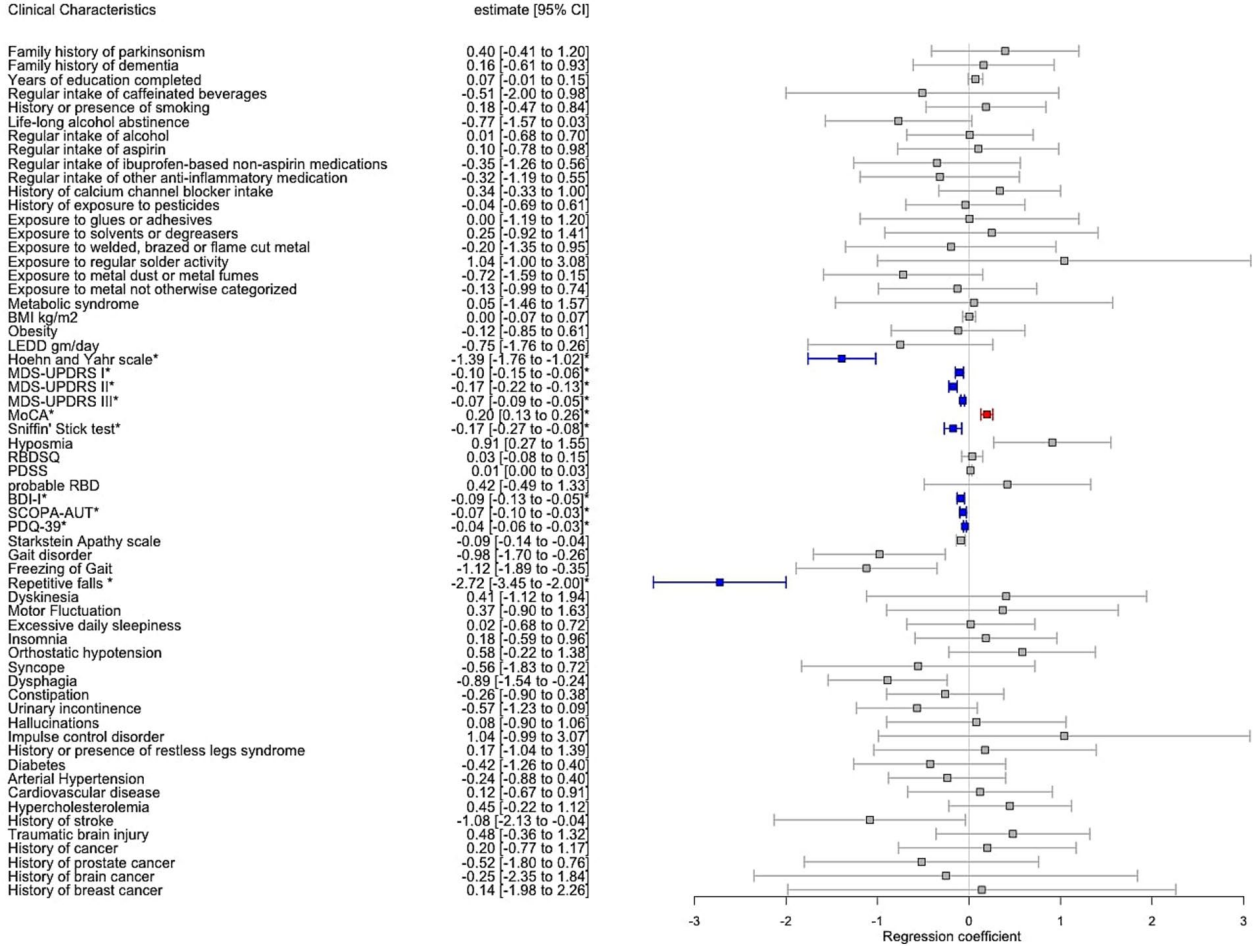
Forest plot with results of multiple regression model including Parkinson’s disease individuals (PD) vs. progressive supranuclear palsy (PSP) adjusted for age at assessment, sex, disease duration and total languages spoken. Estimate corresponds to the regression coefficient with confidence interval 95% (CI). Significant associations after Bonferroni correction for multiple testing were annotated by an asterisk where red colour indicates positive significant association and blue colour negative significant association, respectively, between PD vs. PSP and the clinical variable.

We investigated the similarity of PD with PSP with regard to the outcome variables adjusted for AAA, disease duration, sex and TLS as covariates, with results shown in Figure 4. The linear and logistic regression model indicated PD compared to PSP having significantly lower motor impairment quantified by H&Y [2.21 (0.79) vs. 3.3 (1.26), *p* < 0.001], MDS-UPDRS III [35.2 (16.3) vs. 53.3 (19.5), *p* < 0.001], MDS-UPDRS II [11.5 (8.36) vs. 23.7 (12.3), *p* < 0.001] and a significantly lower frequency of repetitive falls (17.7 vs. 68.6%, *p* < 0.001). In terms of non-motor scales, PD compared to PSP showed a significantly lower burden of depressive symptoms (BDI-I 9.89 [7.06) vs. 16.3 (8.20)*, p <* 0.001], a lower degree of autonomic dysfunction [SCOPA-AUT of 14.9 (8.38) vs. 18.8 (8.82), *p* = 0.01], a lower self-reported burden of non-motor symptoms [MDS-UPDRS I of 10.6 (7.02) vs. 16.5 (8.6), *p* < 0.001]. Furthermore, quality of life was reported significantly higher in PD compared to PSP [PDQ-39 of 39.3 (26.5) vs. 67.8 (28.4), *p* < 0.001]. The olfaction was significantly more affected in PD compared to PSP [mean Sniffin’ Stick score 7.87 (3.52) vs. 9.6 (3.56), *p* = 0.01]. However, when we categorised olfactory sense into hyposmia vs. normosmia, there was no statistical difference in the olfactory capacity between PD vs. PSP (hyposmia 72.9 vs. 53.2%, *p* = 0.41). Finally, global cognition was significantly more affected in PSP compared to PD [MoCA of 20.0 (6.19) vs. 24.3 (4.55), *p* < 0.001].

### Intergroup comparison between PSP and controls

**Figure 5:**
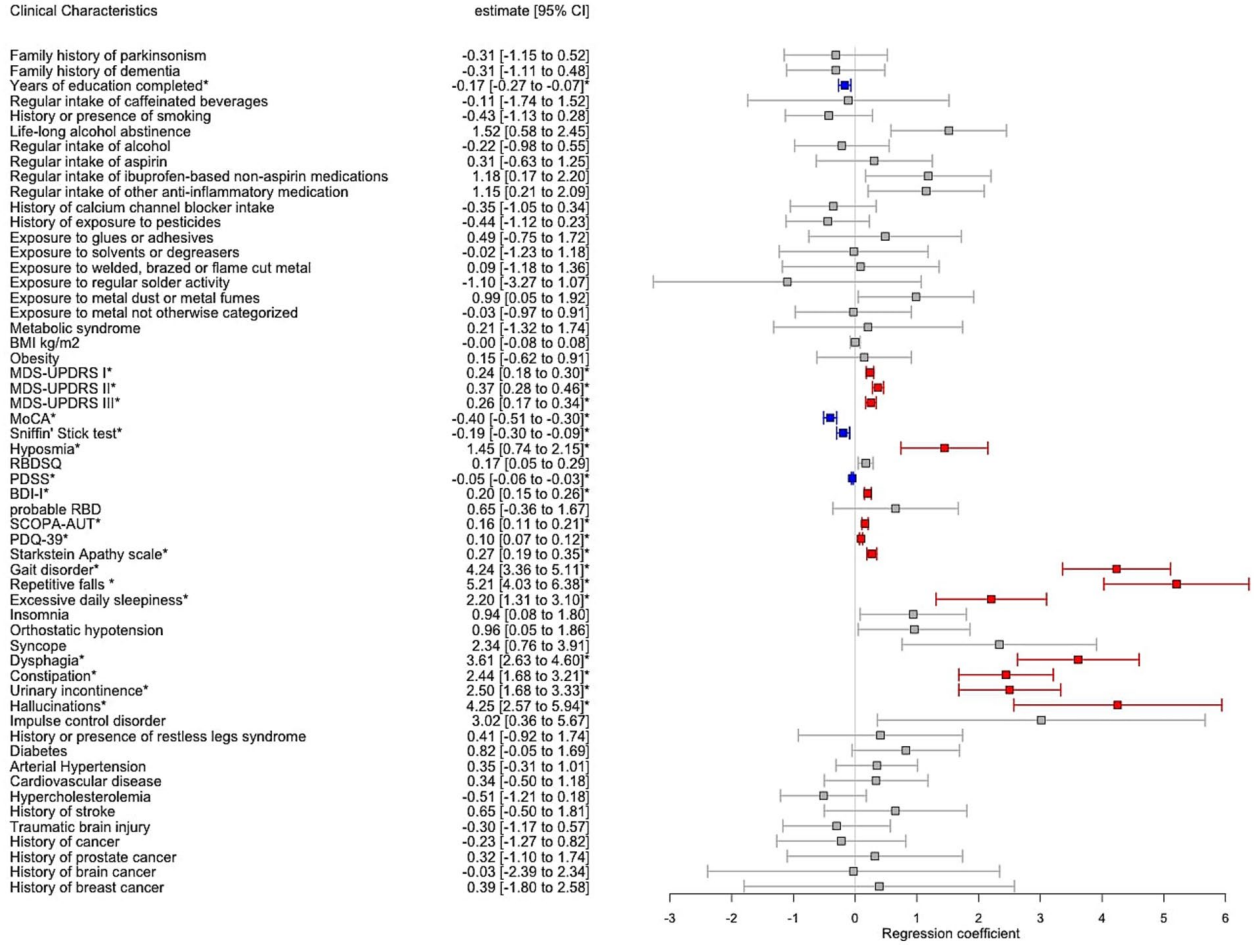
Forest plot with results of multiple regression model including vs. progressive supranuclear palsy (PSP) vs. controls adjusted for age at assessment, sex and total languages spoken. Estimate corresponds to the regression coefficient with confidence interval 95% (CI). Significant associations after Bonferroni correction for multiple testing were annotated by an asterisk where red colour indicates positive significant association and blue colour negative significant association, respectively, between PSP vs. controls and the clinical variable.

In addition to the significant intergroup difference in AAA and sex, we reported a significantly lower education level and less TLS when comparing PSP to controls [11.6 (3.98) vs. 14.3 (3.84), *p* < 0.001 and 2.78 (0.86) vs. 3.5 (0.81), *p* < 0.001, respectively]. Therefore, linear and logistic regression models including 51 patients with PSP and 811 controls were adjusted for AAA, sex and TLS, with results summarized in Figure 5. Of note, olfaction was significantly more affected in PSP vs. controls based on the Sniffin’ Stick test [9.6 (3.56) vs. 12.7 (2.37), *p* = 0.01] with a corresponding higher frequency of hyposmia [53.2 vs. 14.7%, *p* = 0.005]. The remaining clinical outcomes, comorbidities, and environmental exposure significantly associated with PSP vs. controls were listed in Figure 5.

### Sub-analysis of missing data

When investigating the variables with missing data in the (i) pooled patient groups and (ii) controls, 19 variables (out of total number of variables, *n* = 74) were identified with missing data >5% in the patient’s group and none in the control group. Of these, 17 out of 19 variables showed significantly higher disease severity in all three investigated parameters, i.e., higher H&Y, higher MDS-UPDRS III and significantly higher cognitive impairment (measured by MoCA) as indicated in Supplementary Table S3.

## Discussion

### Baseline dataset and comparison to the similar cohort studies

Since the conception of the Luxembourg Parkinson’s study, we have accomplished our recruitment aims to build up one of the largest, monocentric deep-phenotyped cohorts of PD patients globally with parallel recruitment of other forms of neurodegenerative PS and controls (39). It stands out in terms of high sample size, similar male-to-female ratio and AAO when compared with the baseline characteristics of other large recent non-community based PD cohorts as shown in Table 5 [DeNoPA; *De Novo* Parkinson study (40)]; PPMI [Parkinson’s Progression Markers Initiative; (41)]; COPPADIS [COhort of Patients with PArkinson’s DIsease in Spain; (42); Quebec Parkinson Network (43)]; Oxford Parkinson Disease Centre Discovery Cohort [OPDC; (44)] and DEMPARK [Dementia and Parkinson’s disease cohort; (45)]. Additionally, the Luxembourg Parkinson’s study cohort included a wide spectrum of PD as well as atypical PS with *a priori* low recruitment bias by design such as (i) no tertiary referral centre bias, (ii) no participant preselection due to an inclusion criterion with invasive bio-sampling or imaging and (iii) no exclusion criteria on age-limit, cognitive impairment, nor limiting the study inclusion to a certain disease stage (e.g., *de novo* PD or early PD). Such an approach enabled us to achieve a closer look into the real-life spectrum of PD and related disorders. The extensive clinical assessments in the Luxembourg Parkinson’s study allowed for comprehensive analyses of potential risk factor associations for PD and atypical PS in terms of (i) environmental exposures, (ii) dietary habits and medication intake, (iii) comorbidities and (iv) specific disease-related clinical profiles as further discussed.

**Table 5:** Baseline characteristics of exemplary large baseline cohorts including patients with Parkinson’s disease (PD) for cross-comparison with Luxembourg Parkinson’s study baseline dataset.

	PD patients per group (n)	Sex (male in %)	Age at onset at diagnosis in years	Age at assessment in years	Disease duration since diagnosis in years
Luxembourg Parkinson’s study	720	66.5%	62.3 (11.8)	67.3 (10.9)	4.90 (5.16)
DeNoPa	159	66%	not reported*	66 (58–73)	1.25***
PPMI	423	65%	not reported*	61.7 (33–85)	0.56
COPPADIS	694	60.3%	not reported**	62.6 (8.9)	5.5 (4.4)
Quebec Parkinson Network	1,070	64.6%	60.4 (11.0)	68.5 (9.8)	8.9 (6.8)
OPDC Discovery Cohort	490	66.1%	66.1 (9.5)	67.9 (9.3)	1.74 (1.8)
DEMPARK	604	68.2%	not reported**	68.6 (7.9)	6.8 (5.4)

Numbers are reported either in n, mean (standard deviation) or in mean (minimum-maximum value). DeNoPA (De Novo Parkinson study); PPMI (Parkinson’s Progression Markers Initiative); COPPADIS (COhort of Patients with PArkinson’s DIsease in Spain, 2015); OPDC (Oxford Parkinson Disease Centre); DEMPARK (Dementia and Parkinson’s disease cohort). Annotations: *Age at onset (AAO) in PPMI and DeNoPA cohort were not reported as early PD or de novo PD patients were recruited and therefore the AAO was equal/similar to the age at assessment. **COPPADIS and DEMPARK baseline articles did not report on AAO. ***Disease duration in DeNoPA study was reported as disease symptom(s) duration.

### Hyposmia in PSP

In contrast to the reported lack of hyposmia in PSP by Doty et al. (46), we identified a relatively high frequency of hyposmia in PSP (53.2%) following the pattern of the highest frequency of hyposmia in PD > PSP > controls (72.9 vs. 53.2% vs. 14.7%) aligned with more recent results published by Silveira-Moriyama et al. (47). While these studies used different assessments of olfactory function in comparison to our dataset (UPSIT vs. Sniffin’ stick test), the study by Doty et al. included a relatively small number of PSP patients, which substantially limited the statistical power (*n* = 21 vs. Silveira-Moriyama et al. (*n* = 36) vs. our dataset *n* = 51). These findings challenge the traditional view on lack of hyposmia in PSP and might alter the way of using hyposmia as a clinical argument for favouring PD over PSP.

### Sleep-related disorders in PD and PSP

RBD is generally associated with α-synucleinopathies (α-Syn), with high occurrence frequency ranging between 25 and 58% in PD, 70–80% in DLB and up to 90–100% in MSA (48). When comparing to the DeNoPa baseline dataset (40) (*n* = 125 PD, 30% positive for pRBD using the same screening questionnaire and cut-off value), we found comparable frequency of pRBD in our dataset (33.4%). By contrast, tauopathies such as PSP have rarely been reported to present with RBD. Surprisingly, we observed a high frequency of pRBD in PSP (23.3%), supporting the findings of Arnulf et al. (49) and Sixel-Döring et al. (50) who identified a relatively frequent occurrence of RBD in PSP using polysomnographic confirmation. This might suggest that RBD should be considered as a symptom of an underlying pathological process in the brainstem rather than being *exclusively* associated with the pathophysiology of α-Syn. Further examination of brainstem pathology in PSP in this context is warranted to confirm this hypothesis. Among sleep disturbances, RLS is relatively common in the general population, with prevalence ranging between 2.5–15% (51) and observed frequency 3.58% in our control dataset. While former studies on RLS in PSP are scarce and underpowered due to low sample sizes (52), we observed a relatively high frequency of RLS in PSP and even higher in comparison to PD (9.8 vs. 8.76%) though not significant in regression models across all comparisons. Nevertheless, these findings indicate that RLS in PSP may be underdiagnosed, as suggested previously by Gama et al. (where RLS was reported as high as 57% in a small sample of 14 PSP patients) (53).

### Dietary habits and PD

With regard to the dietary habits, we observed a significantly higher frequency of alcohol abstinence in PD compared to controls. This finding concurs with the results of a recent meta-analysis on more than sixty thousand PD patients and nearly 10 million control participants (54). On one hand, the underlying mechanism could be explained by elevated urate levels via ethanol consumption leading to higher pool of antioxidative effect produced by uric acid (55) or possibly by a less pronounced addiction behaviour (in terms of lower risk-seeking and sensation-seeking behaviour) attributed to a premorbid PD personality (56). On the other hand, the cross-sectional set-up of our study cannot claim to imply a causal relationship and the association might be influenced by survival bias or reverse causation as noted in the reference meta-analysis.

### Role of the environmental exposure in PD

In terms of environmental exposure, the population of Luxembourg and the Greater Region is highly exposed to soil contamination by heavy metals (57) and to various chemicals, including pesticides used in agriculture and viticulture (58). As proposed in the *dual hit hypothesis* of PD, two entry points (enteric and olfactory) for toxic and environmental exposure were previously presented as potential contributors to the development of the neurodegenerative process, eventually leading to a dopamine deficit via *substantia nigra* degeneration (59, 60). However, we did not observe a significantly different association of reported pesticide use in PD compared to controls (60.8 vs. 68.2%, *p* = 0.24) in contrast to previous studies (61) nor did we see a significant difference in PD vs. controls when stratifying the pesticide use (at-home and occupational exposure) nor when considering pesticide spraying in the surrounding area (34.9 vs. 35.3%, Supplementary Table S7). We also repeated all pesticide analyses after exclusion of 1^st^, 2^nd^ and 3^rd^ blood relatives and spouses, without observing a change in direction nor in significance of the results (Supplementary Tables S9, S10; Supplementary Figures S2, S3). Compared to the Parkinson Environment Gene study (PEG) focusing on pesticide exposure in highly exposed agricultural areas in California (62), we perceived higher at-home use of pesticides in our dataset vs. PEG [PD 58.9% (403 out of 684) vs. 44.7% (161 out of 360) and controls 66.6% (533 out of 800) vs. 36.5% (302 out of 827)]. In contrast, we identified far lower frequency of occupational use of pesticides in our data vs. PEG [(PD 13% (89 out of 685) vs. 20.6% (74 out of 360) and controls vs. 8.7% (70 out of 802) 13.8% (114 out of 827; for our dataset see Supplementary Tables S7–S10)]. In this context, several relevant aspects and limitations should be taken into account: (i) pesticides, as a general term, are structurally and functionally diverse compounds, not necessarily all linked to the increased risk of neurotoxicity and neurodegeneration [as in the case of rotenone or paraquat (6)], (ii) the questionnaire used in our dataset contained merged groups of pesticides, insecticides, fungicides, herbicides and rodenticides without further granularity, (iii) self-reporting questionnaires are subjected to a recall bias or the exposure might be even present unbeknown to the individual and finally (iv) it might be speculated that in our baseline dataset, AAA in controls was significantly lower than in PD [mean 59.7 (12.1) vs. 67.3 (10.9) years, *p* < 0.001], and thus a proportion of the controls might develop PD in the future. This is a frequent limitation in case–control studies, and we acknowledge it as an inherent limitation of the presented cross-sectional analysis. Nevertheless, the longitudinal design with follow-up of patients and controls will allow us to account for a potential conversion to an overt neurodegenerative disease in the longitudinal data analysis.

### Education and multilingualism in patient and control groups

Interestingly, both PD and PSP, compared to controls, reported significantly less spoken languages [mean 2.83 (1.06) vs. 2.78 (0.86) vs. 3.5 (0.81)] and lower educational level [12.9 (4.08) vs. 11.6 (3.98) vs. 14.3 (3.84) years]. Although it might be speculated that education and multilingualism could play a neuroprotective role in cognitive and neural reserve as previously suggested (63, 64), the difference in multilingualism in our study may partly be explained by the residence of recruited patients compared to controls. The patients joined the Luxembourg Parkinson’s study not only from Luxembourg but also from the surrounding Greater Region (France, Belgium, and Germany), whereas most controls came from Luxembourg. Given a unique multilingual environment in Luxembourg with three official languages (Luxembourgish, German and French), and education system taught in four languages (Luxembourgish, German, French and English), the observed difference in TLS might partially account for this. In the case of higher education in controls vs. patients (PD and PSP), the interpretation of a potential protective effect of education is challenging. And yet, the same trend was observed at other sites such as in the OPDC Discovery cohort [significantly lower education in terms of education years in PD vs. controls 13.7 (3.58) vs. 14.9 (3.49) years (44)]. While access to education, its content and education systems change over time and regions with additional intertwined socioeconomic factors determining the educational level of every individual (65), we argue that the motivation for support of research activities differs between controls and patients. Whereas the motivation for patients with PD and atypical PS to be recruited is mainly for being affected by the disease, the motivation for healthy individuals is mainly due to the higher interest in science and research (20.2% of PD patients vs. 67.5% controls indicated that the main reason for participating in the study was a general interest in science) which could be then linked to more advanced education.

### Cancer in PD

Surprisingly, even in the case of melanoma, with a well-established association between PD and melanoma observed in several epidemiological studies and meta-analyses (66–68), we found no significant association between the investigated comorbidities in all regression models (i.e., PD vs. controls, PD vs. PSP, and PSP vs. controls). However, comparing to the OPDC Discovery cohort with an early-stage PD group at baseline, we found the same trend in the Luxembourg Parkinson’s study dataset reporting higher (but not significant) frequency of melanoma in controls vs. PD (2.8 vs. 2.1% in the OPDC cohort and 1.73 vs. 1.1% in Luxembourg Parkinson’s study respectively). Similarly, we did not observe any significant association between (i) overall frequency of cancer nor (ii) when stratified cancer by type and PD, PSP or controls. Due to a comparable AAA of PD and male-to-female ratio with the baseline PD dataset in OPDC Discovery cohort (*n* = 490), we ascertained a similar frequency of cancer (9.2% in OPDC vs. 12.9% in Luxembourg Parkinson’s study) (44). However, we acknowledge the limitations in our dataset for (i) not systematically capturing the benign vs. malign cancer and (ii) we might expect an underrepresentation of individuals with cancer due to the exclusion criteria in our study, filtering out subjects having active cancer at the time of inclusion.

### Limitations of the study

On this point, several additional limitations should be noted in our study. We observed an unexpectedly high frequency of history of dementia and family history of PS in the control group vs. PD group (34.2 and 34.1% vs. 26.5 and 26.7% respectively). This might be explained by the fact that controls with a family history of a neurodegenerative disorder are generally more motivated to support research and thus participate in our study. Furthermore, the family including the blood relatives accompanying the patients at the research clinic were often recruited in our study based on their interest in advancing research, thus increasing the family history of PS as well as dementia in the control group. Finally, the cross-sectional analysis of disease profiles in PD and PSP could be influenced by a different disease progression rate in both groups, so that the adjustment for disease duration in the regression models might not account for this fact. Indeed, longitudinal studies will be warranted to replicate our findings in order to advance the understanding of these distinct classes of parkinsonian disorders.

### Study strengths

A key strength of our study was the inclusion of PD patients at all disease stages regardless of cognitive status overcoming an important limitation of previous cohort studies. On the one hand, this allows for a more representative picture of the disease profile and serves as an asset of the study, on the other hand, the patients with advanced disease stage or cognitive decline might limit the use and granularity of the self-reported questionnaires especially related to mood, potentially affecting subsets of the results, and increasing the proportion of missing data due to this inherent factor. We addressed this bias in our dataset by the imputation of missing data. As demonstrated, the higher missing data rate in 19 variables (all 19 variables with >5% missing data were self-reported questionnaires) were the trade-off for including patients with atypical PS (typically more severe disease progression in comparison to PD) and more advanced PD patients.

### Conclusion

To our knowledge, the Luxembourg Parkinson’s Study is one of the first pioneering observational studies with deep phenotyping, longitudinally follow-up and biosampling of the patients with PD or atypical PS along with parallel recruitment of controls. It will provide grounds for the patient stratification strategies and further development of personalized medicine approach. The multilevel data generation of the Luxembourg Parkinson’s study has so far provided large-scale genotyping (NeuroChip (69), targeted re-sequencing of *GBA1* gene via PacBio (70) and whole genome sequencing), whole-blood miRNA microarray data, 16S metagenomic data for the gut microbiome, functional models using induced pluripotent stem cells (iPSCs), digital tools / sensor data and brain bank with neuropathological evaluation that continues to contribute to the research field of PD and related disorders. Finally, an annual longitudinal follow-up over up to 9 years (at time of publication) promises to strengthen the understanding of the complex genotype–phenotype interaction, and to identify diagnostic and progression biomarkers unravelling the phenotype variation in patients with neurodegenerative PS.

## Data availability statement

The code for the analytical models and data imputation is publicly available under https://doi.org/10.17881/dy9q-p880. Identical longitudinal biosampling was performed from the baseline visit throughout the follow-up visits including the obligatory sample collection of blood, urine and saliva and voluntary participant’s contribution with stool sample, cerebrospinal fluid (CSF), hair or skin biopsy. The NCER-PD consortium is open for collaboration and exchange of data and biosamples. All data are available upon reasonable request for data or sample(s) according to the national regulations and should be referred to request.ncer-pd@uni.lu.

## Ethics statement

The study was approved by the National Ethics Board in Luxembourg (CNER Ref: 201407/13) and complied with the Declaration of Helsinki. The studies were conducted in accordance with the local legislation and institutional requirements. The participants provided their written informed consent to participate in this study.

## Author contributions

LuP: Conceptualization, Data curation, Investigation, Methodology, Writing – original draft, Writing – review & editing. RR: Formal analysis, Methodology, Writing – review & editing. SG: Methodology, Writing – review & editing. CP: Writing – review & editing. LaP: Writing – review & editing. A-MH: Writing – review & editing. PK: Writing – review & editing. SJ: Writing – review & editing. DM: Writing – review & editing. KA: Writing – review & editing. ET: Writing – review & editing. LV: Writing – review & editing. ES: Writing – review & editing. MGi: Writing – review & editing. OT: Writing – review & editing. SS: Writing – review & editing. ND: Conceptualization, Writing – review & editing. JK: Writing – review & editing. EG: Writing – review & editing. GA: Methodology, Writing – review & editing. ER: Writing – review & editing. MP: Writing – review & editing. MV: Methodology, Writing – review & editing. PM: Writing – review & editing. MGa: Conceptualization, Writing – review & editing. VS: Conceptualization, Writing – review & editing. RK: Conceptualization, Funding acquisition, Investigation, Methodology, Writing – review & editing.

## Group members of [NCER-PD Consortium]

Geeta Acharya^2^, Gloria Aguayo^2^, Myriam Alexandre^2^, Muhammad Ali^1^, Wim Ammerlann^2^, Giuseppe Arena^1^, Rudi Balling^1^, Michele Bassis^1^, Roxane Batutu^3^, Katy Beaumont^2^, Regina Becker^1^, Camille Bellora^2^, Guy Berchem^3^, Daniela Berg^11^, Alexandre Bisdorff^5^, Ibrahim Boussaad^1^, Kathrin Brockmann^11^, Jessica Calmes^2^, Lorieza Castillo^2^, Gessica Contesotto^2^, Nancy De Bremaeker^3^, Nico Diederich^3^, Rene Dondelinger^5^, Daniela Esteves^2^, Guy Fagherazzi^2^, Jean-Yves Ferrand^2^, Manon Gantenbein^2^, Thomas Gasser^11^, Piotr Gawron^1^, Soumyabrata Ghosh^1^, Marijus Giraitis^2,3^, Enrico Glaab^1^, Elisa Gómez De Lope^1^, Jérôme Graas^2^, Mariella Graziano^17^, Valentin Groues^1^, Anne Grünewald^1^, Wei Gu^1^, Gaël Hammot^2^, Anne-Marie Hanff^2,20,21^, Linda Hansen^1,3^, Michael Heneka^1^, Estelle Henry^2^, Sylvia Herbrink^6^, Sascha Herzinger^1^, Michael Heymann^2^, Michele Hu^8^, Alexander Hundt^2^, Nadine Jacoby^18^, Jacek Jaroslaw Lebioda^1^, Yohan Jarosz^1^, Sonja Jónsdóttir^2^, Quentin Klopfenstein^1^, Jochen Klucken^1,2,3^, Rejko Krüger^1,2,3^, Pauline Lambert^2^, Zied Landoulsi^1^, Roseline Lentz^7^, Inga Liepelt^11^, Robert Liszka^14^, Laura Longhino^3^, Victoria Lorentz^2^, Paula Cristina Lupu^2^, Tainá M. Marques^1^, Clare Mackay^10^, Walter Maetzler^15^, Katrin Marcus^13^, Guilherme Marques^2^, Patricia Martins Conde^1^, Patrick May^1^, Deborah Mcintyre^2^, Chouaib Mediouni^2^, Francoise Meisch^1^, Myriam Menster^2^, Maura Minelli^2^, Michel Mittelbronn^1,4^, Brit Mollenhauer^12^, Friedrich Mühlschlegel^4^, Romain Nati^3^, Ulf Nehrbass^2^, Sarah Nickels^1^, Beatrice Nicolai^3^, Jean-Paul Nicolay^19^, Fozia Noor^2^, Marek Ostaszewski^1^, Clarissa P. C. Gomes^1^, Sinthuja Pachchek^1^, Claire Pauly^1,3^, Laure Pauly^2, 20^, Lukas Pavelka^1,2,3^, Magali Perquin^2^, Nancy E. Ramia^1^, Rosalina Ramos Lima^2^, Armin Rauschenberger^1^, Rajesh Rawal^1^, Dheeraj Reddy Bobbili^1^, Kirsten Roomp^1^, Eduardo Rosales^2^, Isabel Rosety^1^, Estelle Sandt^2^, Stefano Sapienza^1^, Venkata Satagopam^1^, Margaux Schmitt^2^, Sabine Schmitz^1^, Reinhard Schneider^1^, Jens Schwamborn^1^, Amir Sharify^2^, Ekaterina Soboleva^1^, Kate Sokolowska^2^, Hermann Thien^2^, Elodie Thiry^1,3^, Rebecca Ting Jiin Loo^1^, Christophe Trefois^1^, Johanna Trouet^2^, Olena Tsurkalenko^2^, Michel Vaillant^2^, Mesele Valenti^2^, Gilles Van Cutsem^1,3^, Carlos Vega^1^, Liliana Vilas Boas^3^, Maharshi Vyas^1^, Richard Wade-Martins^9^, Paul Wilmes^1^, Evi Wollscheid-Lengeling^1^, Gelani Zelimkhano^3^.

^1^Luxembourg Centre for Systems Biomedicine, University of Luxembourg, Esch-sur-Alzette, Luxembourg.

^2^Luxembourg Institute of Health, Strassen, Luxembourg.

^3^Centre Hospitalier de Luxembourg, Strassen, Luxembourg.

^4^Laboratoire National de Santé, Dudelange, Luxembourg.

^5^Centre Hospitalier Emile Mayrisch, Esch-sur-Alzette, Luxembourg.

^6^Centre Hospitalier du Nord, Ettelbrück, Luxembourg.

^7^Parkinson Luxembourg Association, Leudelange, Luxembourg.

^8^Oxford Parkinson’s Disease Centre, Nuffield Department of Clinical Neurosciences, University of Oxford, Oxford, United Kingdom.

^9^Oxford Parkinson’s Disease Centre, Department of Physiology, Anatomy and Genetics, University of Oxford, South Parks Road, Oxford, United Kingdom.

^10^Oxford Centre for Human Brain Activity, Wellcome Centre for Integrative Neuroimaging, Department of Psychiatry, University of Oxford, Oxford, United Kingdom.

^11^Center of Neurology and Hertie Institute for Clinical Brain Research, Department of Neurodegenerative Diseases, University Hospital Tübingen, Germany.

^12^Paracelsus-Elena-Klinik, Kassel, Germany.

^13^ Ruhr-University of Bochum, Bochum, Germany.

^14^Westpfalz-Klinikum GmbH, Kaiserslautern, Germany.

^15^Department of Neurology, University Medical Center Schleswig-Holstein, Kiel, Germany.

^16^Department of Neurology Philipps, University Marburg, Marburg, Germany.

^17^Association of Physiotherapists in Parkinson’s Disease Europe, Esch-sur-Alzette, Luxembourg.

^18^Private Practice, Ettelbruck, Luxembourg.

^19^ Private Practice, Luxembourg, Luxembourg.

^20^Faculty of Science, Technology and Medicine, University of Luxembourg, Esch-sur-Alzette, Luxembourg.

^21^Department of Epidemiology, CAPHRI School for Public Health and Primary Care, Maastricht University Medical Centre+, Maastricht, Netherlands.
