# Supplementary material for: Luxembourg Parkinson’s study -comprehensive baseline analysis of Parkinson’s disease and atypical parkinsonism
Source: Front Neurol. 2023 Dec 19;14:1330321. doi: 10.3389/fneur.2023.1330321 (PMC10763250; doi:10.3389/fneur.2023.1330321)
Supplement: Supplementary file 1 [file Data_Sheet_1.pdf]

Supplementary material

Additional definitions for clinical characteristics, symptoms, and scales

The clinical scales were previously described in a methodological article for the Luxembourg Parkinson's Study [1]. To precise, Movement Disorder Society (MDS) - Unified Parkinson's disease Rating Scale (MDS-UPDRS I-IV) and Scales for Outcomes in Parkinson's Disease-Autonomic questionnaire (SCOPA-AUT) were used under the license number (14017_ND). The Hoehn and Yahr scale (H&Y) corresponds to the modified version of the scale [2]. Assessment of sleep quality was done via Parkinson's Disease Sleep Scale (PDSS [3]). Definition of constipation corresponds to the diagnostic criteria ROME III and information was acquired in a semi-structured interview [4]. Quality of life was assessed via Parkinson's disease Questionnaire 39 (PDQ-39 [5]). Depression symptoms were reflected by Beck Depression Inventory Version I (BDI-I [6]) Cognitive performance was assessed via Montreal Cognitive Assessment (MoCA [7]). Non-Motor Symptoms Scale (NMSS) was used first published and validated in 2007 [8]. Apathy was assessed via validated Starkstein Apathy scale [9]. Presence of recurrent orthostatic hypotension was assessed using a semi-structured interview inquiring for the symptoms of orthostatic hypotension, i.e., faintness, dizziness, light-headedness, vertigo, hearing disturbance, visual disturbance or syncope following the tilting, standing up or after a long standing relieved by sitting down or laying down. All symptoms included in the analysis (listed in Table 3) were assessed during a semi-structured interview of the participant and/or the participant's proxy with a study physician and refer to the current motor and non-motor symptoms at the time of assessment.

Data monitoring process

To ensure that the study was conducted according to the planned protocol and amendments in compliance with Good Clinical Practice (GCP) and relevant legislation, monitoring activities were set up according to a monitoring plan agreed by the Principal Investigator. External study monitor was appointed to execute and supervise the data monitoring process in accordance with the data monitoring plan. This process was applied to ensure that data was accurate, complete and verifiable from source documents along with the protection of rights and well-being of the participants. The monitoring took place at the Parkinson's Research Clinic (PRC) for patient group and at the Clinical and Epidemiological Investigation Center (CIEC) for control group. The monitoring allowed to calculate the recruitment rate per site, to check the availability of study documents to participants (informed consent forms, study information sheet, case-report-form (CRF) and questionnaires) in order to ensure the most recent versions of the data capture forms were used. In addition, the source data

verification was performed to check consistency between source data and data that are captured in the electronic CRF platform in REDCap.

Supplementary References

1. Hipp G, Vaillant M, Diederich NJ, et al. The Luxembourg Parkinson's Study: A Comprehensive Approach for Stratification and Early Diagnosis. *Front Aging Neurosci*. 2018;10:326. doi:10.3389/fnagi.2018.00326

2. Goetz CG, Poewe W, Rascol O, et al. *Movement* Disorder Society Task Force report on the Hoehn and Yahr staging scale: Status and recommendations The *Movement* Disorder Society Task Force on rating scales for Parkinson's disease. *Mov Disord*. 2004;19(9):1020-1028. doi:10.1002/mds.20213

3. Chaudhuri KR. The Parkinson's disease sleep scale: a new instrument for assessing sleep and nocturnal disability in Parkinson's disease. *J Neurol Neurosurg Psychiatry*. 2002;73(6):629-635. doi:10.1136/jnnp.73.6.629

4. Longstreth GF, Thompson WG, Chey WD, Houghton LA, Mearin F, Spiller RC. Functional Bowel Disorders. *Gastroenterology*. 2006;130(5):1480-1491. doi:10.1053/j.gastro.2005.11.061

5. Peto V, Jenkinson C, Fitzpatrick R, Greenhall R. The development and validation of a short measure of functioning and well being for individuals with Parkinson's disease. *Qual Life Res*. 1995;4(3):241-248. doi:10.1007/BF02260863

6. Beck AT. An Inventory for Measuring Depression. *Arch Gen Psychiatry*. 1961;4(6):561. doi:10.1001/archpsyc.1961.01710120031004

7. Nasreddine ZS, Phillips NA, BÃ©dirian V, et al. The Montreal Cognitive Assessment, MoCA: A Brief Screening Tool For Mild Cognitive Impairment: MOCA: A BRIEF SCREENING TOOL FOR MCI. *J Am Geriatr Soc*. 2005;53(4):695-699. doi:10.1111/j.1532-5415.2005.53221.x

8. Chaudhuri KR, Martinez-Martin P, Brown RG, et al. The metric properties of a novel non-motor symptoms scale for Parkinson's disease: Results from an international pilot study. *Mov Disord*. 2007;22(13):1901-1911. doi:10.1002/mds.21596

9. Reliability, validity, and clinical correlates of apathy in Parkinson's disease. *J Neuropsychiatry Clin Neurosci*. 1992;4(2):134-139. doi:10.1176/jnp.4.2.134

SUPPLEMENTARY TABLES AND FIGURES

Supplementary Table S1. Stratification of Parkinson's disease (PD) and control group by sex and age at assessment (AAA)

AAA (years)	18-25	26-35	36-45	46-55	56-65	>65
	**Female**	**Female**	**Female**	**Female**	**Female**	**Female**
CONTROL	4	15	31	73	130	135
PD	0	0	7	33	52	149
Total	4	15	38	106	182	284
	**Male**	**Male**	**Male**	**Male**	**Male**	**Male**
CONTROL	1	14	29	95	117	167
PD	1	1	10	56	109	302
Total	2	15	39	151	226	469

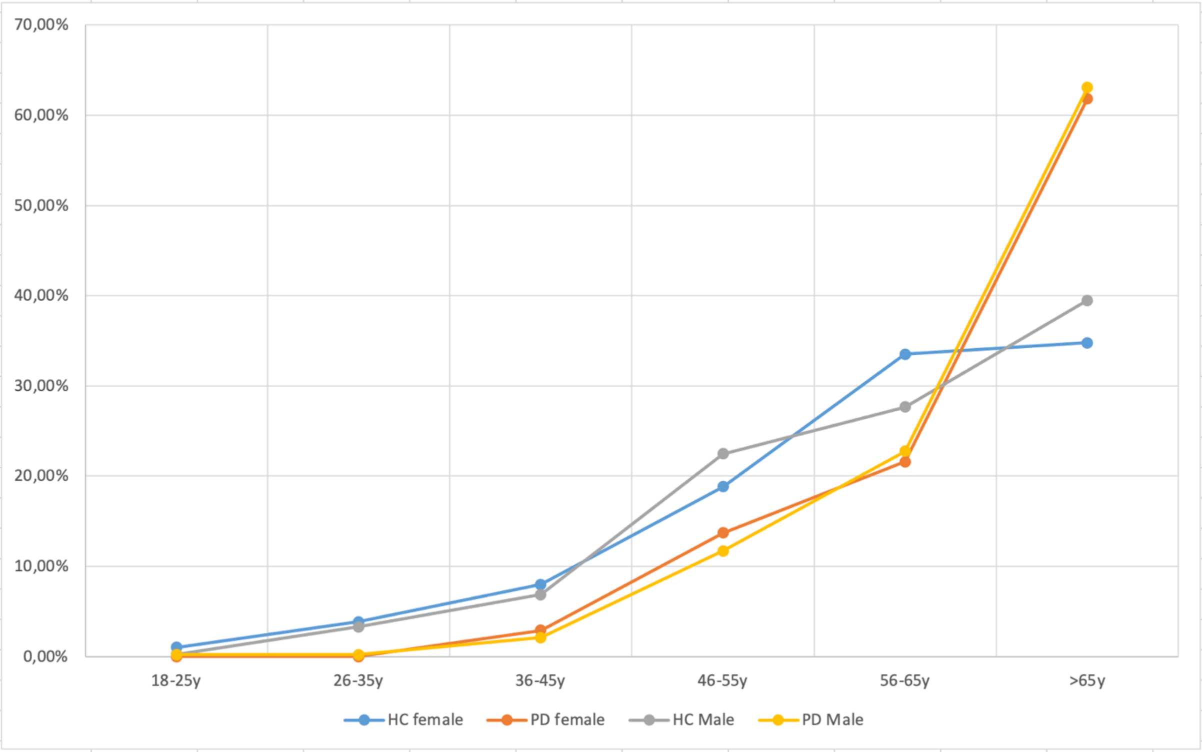

Supplementary Figure S1. Stratification of Parkinson's disease (PD) and control group by sex and age at assessment (AAA) with overall proportion (in %) to the diagnostic group (based on Supplementary Table 1).

Supplementary Table S2. Missing data per variable grouped by diagnostic status in baseline dataset of Luxembourg Parkinson's Study.

	PD (n=720)		Controls (n=811)		PSP (n=51)		Other parkinsonism (n=66)	
	**missing (n)**	**%**	**missing (n)**	**%**	**missing (n)**	**%**	**missing (n)**	**%**
Family history of parkinsonism	1	0.14	2	0.25	2	3.92	0	0
Family history of dementia	7	0.97	6	0.74	2	3.92	0	0
Sex	0	0	0	0	0	0	0	0
Age at baseline assessment	0	0	0	0	0	0	0	0
Disease duration since diagnosis	1	0.14	-	-	0	0	0	0
Age at onset	1	0.14	-	-	0	0	0	0
Years of education	5	0.69	7	0.86	0	0	1	1.52
Total languages spoken	0	0	0	0	0	0	0	0
Regular intake of caffeinated beverages	34	4.72	9	1.11	3	5.88	4	6.06
History or presence of smoking	37	5.14	9	1.11	3	5.88	4	6.06
Life-long alcohol abstinence	56	7.78	28	3.45	6	11.77	9	13.64
Regular intake of alcohol	47	6.53	22	2.71	4	7.84	6	9.09
Regular intake of aspirin	50	6.94	17	2.10	7	13.73	10	15.15
Regular intake of ibuprofen-based non-aspirin medication	64	8.89	30	3.70	8	15.69	13	19.70
Regular intake of other anti-inflammatory drugs	61	8.47	27	3.33	8	15.69	8	12.12
History of calcium channel blocker intake	53	7.36	20	2.47	7	13.73	7	10.61
History of exposure to pesticides	34	4.72	9	1.11	3	5.88	4	6.06
Exposure to glues or adhesives	57	7.92	25	3.08	6	11.77	7	10.61
Exposure to solvents or degreasers	60	8.33	30	3.70	6	11.77	7	10.61
Exposure to welded brazed flame or flame cut metal	43	5.97	13	1.60	4	7.84	5	7.58
Exposure to regular solder activity	42	5.83	12	1.48	6	11.77	5	7.58
Exposure to metal dust or metal fumes	55	7.64	18	2.22	4	7.84	5	7.58
Exposure to metal not otherwise categorized	74	10.28	39	4.81	6	11.77	6	9.09
Metabolic syndrome	0	0	1	0.12	1	1.96	1	1.52
BMI	22	3.06	2	0.25	6	11.77	5	7.58
Obesity	22	3.06	2	0.25	6	11.77	5	7.58
LEDD	0	0	0	0	0	0	0	0
Hoehn&Yahr stage	3	0.42	2	0.25	0	0	0	0
MDS-UPDRS I	23	3.19	10	1.23	2	3.92	2	3.03
MDS-UPDRS II	18	2.5	7	0.86	2	3.92	2	3.03
MDS-UPDRS III	15	2.08	23	2.84	0	0	0	0
MDS-UPDRS IV	8	1.11	12	1.48	1	1.96	1	1.52
MoCA	17	2.36	2	0.25	7	13.73	4	6.06
Sniffin' Stick test	18	2.5	3	0.37	4	7.84	2	3.03
NMSS*	92	12.78	32	3.95	11	21.57	11	16.67
Hyposmia	18	2.5	3	0.37	4	7.84	2	3.03
RBDSQ	64	8.89	30	3.70	8	15.69	8	12.12
pRBD	64	8.89	30	3.70	8	15.69	8	12.12
PDSS	58	8.06	25	3.08	7	13.73	9	13.64
BDI-I	43	5.97	19	2.34	8	15.69	6	9.09

SCOPA-AUT	49	6.80	27	3.33	10	19.61	7	10.61
PDQ 39	70	9.72	29	3.58	6	11.77	10	15.15
Starkstein Apathy scale	59	8.19	26	3.21	7	13.73	8	12.12
Gait Disoder	1	0.14	0	0	0	0	0	0
Freezing of Gait	1	0.14	0	0	0	0	0	0
Falls	1	0.14	0	0	0	0	0	0
Dyskinesia	1	0.14	0	0	0	0	0	0
Motor Fluctuation	1	0.14	0	0	0	0	0	0
Excessive daily sleepiness	1	0.14	0	0	0	0	0	0
Insomnia	1	0.14	0	0	0	0	0	0
Orthostatic hypotension	1	0.14	0	0	0	0	0	0
Syncope	1	0.14	0	0	0	0	0	0
Dysphagia	1	0.14	0	0	0	0	0	0
Constipation	1	0.14	0	0	0	0	0	0
Urinary incontinence	1	0.14	0	0	0	0	0	0
Hallucinations	1	0.14	0	0	0	0	0	0
Impulse control disorder	1	0.14	0	0	0	0	0	0
History or presence of restless legs syndrome	1	0.14	0	0	0	0	0	0
Diabetes	1	0.14	0	0	0	0	0	0
Arterial hypertension	1	0.14	0	0	0	0	0	0
Cardiovascular disease	1	0.14	0	0	0	0	0	0
Hypercholesterolemia	1	0.14	0	0	0	0	0	0
History of epileptic seizures	1	0.14	0	0	0	0	0	0
History of stroke	1	0.14	0	0	0	0	0	0
Traumatic brain injury	1	0.14	0	0	0	0	0	0
History of cancer	1	0.14	0	0	0	0	0	0
History of melanoma	1	0.14	0	0	0	0	0	0
History of prostate cancer	1	0.14	0	0	0	0	0	0
History of brain tumor	1	0.14	0	0	0	0	0	0
History of breast cancer	1	0.14	0	0	0	0	0	0
History of cancer categorized	1	0.14	0	0	0	0	0	0

Annotation * Non-motor Symptom Scale (NMSS) was introduced after the start of the study recruitment, therefore the missing values were not missing at random (not imputed as not used for the regression analysis).

Supplementary table S3. Subanalysis of variables with missing data >5% in the Luxembourg Parkinson's study baseline dataset. All patients with parkinsonism were merged (n=784) and the age and surrogate markers of disease severity were compared per variable after exclusion of the individuals having at least one not testable (=non-imputable) value. Significant findings were annotated by asterisk at Bonferroni adjusted *p*-level.

			Patients with parkinsonism (merged) **Non-missing data** Mean (SD)	Patients with parkinsonism (merged) **Missing data** Mean (SD)	OR [CI 95%]	Bonferroni adjusted p-value
1.	Exposure to metal not otherwise categorized		N=707	N=77		
		Age at assessment (years)	67.5 (10.4)	66.9 (11.5)	0.99 [0.97;1.02]	1
		MDS UPDRS III*	35.4 (16.2)	41.2 (18.7)	1.02 [1.01;1.03]	0,042
		H&Y	2.24 (0.81)	2.47 (1.00)	1.34 [1.04;1.72]	0,209
		MoCA*	24.1 (4.53)	22.5 (5.12)	0.94 [0.90;0.98]	0,04
2.	Regular intake of ibuprofen-based non-aspirin medication		N=709	N=75		
		Age at assessment (years)*	67.0 (10.4)	71.8 (10.8)	1.05 [1.02;1.08]	0,002
		MDS UPDRS III*	35.2 (16.2)	43.7 (18.3)	1.03 [1.02;1.04]	0,001
		H&Y*	2.23 (0.81)	2.63 (1.01)	1.62 [1.27;2.06]	0,005
		MoCA*	24.3 (4.45)	21.3 (5.24)	0.89 [0.85;0.93]	p<0.001
3.	PDQ-39		N=710	N=74		
		Age at assessment (years)	67.2 (10.4)	70.4 (11.4)	1.03 [1.01;1.06]	0,082
		MDS UPDRS III*	35.1 (16.1)	44.8 (18.6)	1.03 [1.02;1.05]	p<0.001
		H&Y*	2.22 (0.78)	2.74 (1.16)	1.81 [1.43;2.30]	0,001
		MoCA*	24.3 (4.41)	21.3 (5.63)	0.89 [0.86;0.93]	p<0.001
4.	Probable RBD		N=716	N=68		
		Age at assessment (years)*	67.1 (10.3)	71.3 (11.9)	1.04 [1.02;1.07]	0,025
		MDS UPDRS III*	35.1 (16.0)	45.0 (19.2)	1.03 [1.02;1.05]	p<0.001
		H&Y*	2.22 (0.79)	2.74 (1.14)	1.81 [1.42;2.31]	0,002
		MoCA*	24.2 (4.43)	21.2 (5.59)	0.89 [0.85;0.93]	p<0.001
5.	RBDSQ		N=716	N=68		
		Age at assessment (years)*	67.1 (10.3)	71.3 (11.9)	1.04 [1.02;1.07]	0,025
		MDS UPDRS III*	35.1 (16.0)	45.0 (19.2)	1.03 [1.02;1.05]	p<0.001
		H&Y*	2.22 (0.79)	2.74 (1.14)	1.81 [1.42;2.31]	0,002
		MoCA*	24.2 (4.43)	21.2 (5.59)	0.89 [0.85;0.93]	p<0.001
6.	Starkstein Apathy scale		N=718	N=66		
		Age at assessment (years)	67.3 (10.4)	69.6 (11.5)	1.02 [1.00;1.05]	0,495
		MDS UPDRS III*	35.1 (16.2)	45.8 (17.7)	1.04 [1.02;1.05]	p<0.001
		H&Y*	2.22 (0.79)	2.70 (1.15)	1.72 [1.34;2.20]	0,007
		MoCA*	24.3 (4.37)	20.6 (5.71)	0.87 [0.83;0.91]	p<0.001
7.	PDSS		N=719	N=65		
		Age at assessment (years)	67.2 (10.4)	70.1 (11.4)	1.03 [1.00;1.06]	0,207
		MDS UPDRS III*	35.2 (16.1)	45.0 (19.1)	1.03 [1.02;1.05]	0,001
		H&Y*	2.23 (0.80)	2.64 (1.09)	1.61 [1.25;2.08]	0,017
		MoCA*	24.3 (4.43)	20.9 (5.50)	0.88 [0.84;0.93]	p<0.001
8.	Regular intake of other anti-inflammatory drugs		N=719	N=65		
		Age at assessment (years)	67.3 (10.4)	69.8 (11.8)	1.02 [1.00;1.05]	0,423
		MDS UPDRS III*	35.1 (16.2)	45.5 (18.1)	1.03 [1.02;1.05]	p<0.001
		H&Y*	2.22 (0.81)	2.74 (0.99)	1.79 [1.40;2.30]	p<0.001

		MoCA*	24.2 (4.49)	21.3 (5.19)	0.90 [0.86;0.94]	p<0.001
9.	Life-long alcohol abstinence		N=720	N=64		
		Age at assessment (years)	67.3 (10.4)	69.4 (11.6)	1.02 [0.99;1.05]	0,707
		MDS UPDRS III*	35.3 (16.3)	43.1 (17.7)	1.03 [1.01;1.04]	0,005
		H&Y*	2.24 (0.82)	2.55 (0.94)	1.47 [1.13;1.91]	0,046
		MoCA*	24.1 (4.54)	22.1 (5.08)	0.92 [0.88;0.97]	0,013
10.	Exposure to solvents or degreasers		N=723	N=61		
		Age at assessment (years)	67.4 (10.4)	68.4 (11.3)	1.01 [0.98;1.04]	1
		MDS UPDRS III*	35.4 (16.4)	42.6 (17.7)	1.02 [1.01;1.04]	0,012
		H&Y*	2.23 (0.82)	2.62 (0.98)	1.58 [1.21;2.05]	0,014
		MoCA*	24.1 (4.53)	22.2 (5.24)	0.93 [0.88;0.97]	0,027
11.	History of calcium channel blocker intake		N=725	N=59		
		Age at assessment (years)	67.2 (10.4)	70.7 (11.1)	1.03 [1.01;1.06]	0,089
		MDS UPDRS III	35.5 (16.3)	41.8 (18.6)	1.02 [1.01;1.04]	0,057
		H&Y*	2.23 (0.81)	2.64 (1.02)	1.61 [1.24;2.10]	0,014
		MoCA*	24.2 (4.52)	21.7 (5.19)	0.91 [0.87;0.96]	0,003
12.	Exposure to glues or adhesives		N=725	N=59		
		Age at assessment (years)	67.3 (10.4)	69.5 (11.2)	1.02 [0.99;1.05]	0,581
		MDS UPDRS III*	35.2 (16.3)	45.0 (17.4)	1.03 [1.02;1.05]	p<0.001
		H&Y*	2.23 (0.81)	2.73 (0.97)	1.76 [1.36;2.28]	0,001
		MoCA*	24.2 (4.51)	21.8 (5.27)	0.91 [0.87;0.96]	0,005
13.	Regular intake of aspirin		N=726	N=58		
		Age at assessment (years)	67.2 (10.4)	70.7 (11.8)	1.03 [1.01;1.06]	0,137
		MDS UPDRS III*	35.0 (16.1)	47.8 (18.3)	1.04 [1.03;1.06]	p<0.001
		H&Y*	2.22 (0.79)	2.84 (1.11)	1.96 [1.52;2.53]	p<0.001
		MoCA*	24.2 (4.47)	21.0 (5.32)	0.89 [0.85;0.93]	p<0.001
14.	Exposure to metal dust or metal fumes		N=727	N=57		
		Age at assessment (years)	67.3 (10.5)	69.4 (10.7)	1.02 [0.99;1.05]	0,646
		MDS UPDRS III*	35.3 (16.1)	45.2 (19.1)	1.03 [1.02;1.05]	0,001
		H&Y*	2.23 (0.80)	2.68 (1.09)	1.68 [1.29;2.19]	0,013
		MoCA*	24.2 (4.50)	21.5 (5.30)	0.90 [0.86;0.95]	0,002
15.	SCOPA-AUT		N=730	N=54		
		Age at assessment (years)	67.4 (10.4)	68.6 (12.3)	1.01 [0.98;1.04]	1
		MDS UPDRS III*	35.2 (16.1)	46.7 (18.4)	1.04 [1.02;1.05]	p<0.001
		H&Y*	2.23 (0.80)	2.78 (1.11)	1.84 [1.41;2.39]	0,003
		MoCA*	24.2 (4.46)	21.1 (5.66)	0.89 [0.85;0.94]	0,001
16.	Regular intake of alcohol		N=734	N=50		
		Age at assessment (years)	67.3 (10.4)	69.5 (11.3)	1.02 [0.99;1.05]	0,819
		MDS UPDRS III*	35.3 (16.1)	45.7 (19.4)	1.03 [1.02;1.05]	0,002
		H&Y*	2.23 (0.81)	2.78 (1.05)	1.83 [1.39;2.40]	0,003
		MoCA*	24.2 (4.48)	21.1 (5.62)	0.89 [0.85;0.94]	0,002
17.	Exposure to welded brazed flame or flame cut metal		N=739	N=45		
		Age at assessment (years)	67.3 (10.4)	70.2 (11.9)	1.03 [1.00;1.06]	0,483
		MDS UPDRS III*	35.4 (16.3)	45.5 (18.2)	1.03 [1.02;1.05]	0,003
		H&Y*	2.24 (0.82)	2.74 (1.00)	1.76 [1.32;2.34]	0,006
		MoCA*	24.1 (4.54)	21.3 (5.03)	0.90 [0.85;0.95]	0,002

18.	BDI-I	N=740	N=44		
	Age at assessment (years)	67.3 (10.4)	70.2 (11.3)	1.03 [1.00;1.06]	0,404
	MDS UPDRS III*	35.4 (16.4)	46.0 (15.7)	1.04 [1.02;1.05]	p<0.001
	H&Y*	2.24 (0.83)	2.66 (0.92)	1.61 [1.20;2.17]	0,02
	MoCA*	24.2 (4.46)	20.0 (5.35)	0.86 [0.82;0.91]	p<0.001
19.	Exposure to regular solder activity	N=740	N=44		
	Age at assessment (years)	67.4 (10.4)	69.6 (11.6)	1.02 [0.99;1.05]	0,904
	MDS UPDRS III*	35.3 (16.2)	47.8 (18.2)	1.04 [1.02;1.06]	p<0.001
	H&Y*	2.23 (0.81)	2.83 (1.06)	1.90 [1.43;2.53]	0,002
	MoCA*	24.2 (4.50)	20.8 (5.29)	0.89 [0.84;0.93]	0,001

Supplementary Table S4. Sociodemographic characteristics and environmental exposure in Luxembourg Parkinson's study baseline dataset: diagnostic groups that were not analysed for the reason of small sample size.

	DLB[1] Mean (SD) or YES/NO (%)	MSA[2] Mean (SD) or YES/NO (%)	Unspecified PS[3] Mean (SD) or YES/NO (%)	Rare PS[4] Mean (SD) or YES/NO (%)	Vascular PS[5] Mean (SD) or YES/NO (%)
Individuals per group	19	12	18	3	14
Sex (male)	13/6 (68.4%)	5/7 (41.7%)	13/5 (72.2%)	2/1 (66.7%)	10/4 (71.4%)
Age at baseline assessment (years)	77.8 (5.58)	65.9 (12.0)	69.4 (7.03)	63.8 (13.4)	73.8 (5.57)
Age at onset (years)	75.6 (5.64)	62.3 (12.6)	66.9 (8.51)	59.7 (11.6)	69.9 (6.37)
Family history of parkinsonism	2/17 (10.5%)	2/10 (16.7%)	5/13 (27.8%)	0/3 (0.00%)	4/10 (28.6%)
Family history of dementia	8/11 (42.1%)	4/8 (33.3%)	6/12 (33.3%)	0/3 (0.00%)	1/13 (7.14%)
Years of education completed	9.50 (4.44)	12.2 (4.88)	13.6 (5.50)	11.7 (1.53)	11.8 (4.28)
Total languages spoken	3.05 (0.85)	3.25 (0.97)	3.28 (0.75)	2.67 (1.53)	3.07 (0.73)
Regular intake of caffeinated beverages	16/2 (88.9%)	10/0 (100%)	18/0 (100%)	2/1 (66.7%)	12/1 (92.3%)
History or presence of smoking	11/7 (61.1%)	6/4 (60.0%)	14/4 (77.8%)	3/0 (100%)	6/7 (46.2%)
Life-long alcohol abstinence	5/13 (27.8%)	4/5 (44.4%)	2/13 (13.3%)	0/3 (0%)	1/11 (8.3%)
Regular intake of alcohol	13/5 (72.2%)	5/5 (50.0%)	13/4 (76.5%)	2/1 (66.7%)	8/4 (66.7%)
Regular intake of aspirin	4/11 (26.7%)	0/9 (0.00%)	5/13 (27.8%)	0/3 (0.00%)	6/5 (54.5%)
Regular intake of ibuprofen-based non-aspirin medications	3/11 (21.4%)	1/8 (11.1%)	5/11 (31.2%)	0/3 (0.00%)	3/8 (27.3%)
Regular intake of other anti-inflammatory medication	6/11 (35.3%)	2/8 (20.0%)	3/14 (17.6%)	0/3 (0.00%)	1/10 (9.09%)
History of calcium channel blocker intake	8/9 (47.1%)	4/6 (40.0%)	9/8 (52.9%)	1/2 (33.3%)	9/3 (75.0%)
History of exposure to pesticides	13/5 (72.2%)	2/8 (20.0%)	12/6 (66.7%)	3/0 (100%)	8/5 (61.5%)
Exposure to glues or adhesives	3/15 (16.7%)	0/10 (0.00%)	2/14 (12.5%)	1/2 (33.3%)	2/10 (16.7%)
Exposure to metal dust or metal fumes	7/11 (38.9%)	1/9 (10.0%)	3/14 (17.6%)	0/3 (0.00%)	1/12 (7.69%)
Exposure to regular solder activity	3/15 (16.7%)	0/10 (0.00%)	1/16 (5.88%)	0/3 (0.00%)	0/13 (0.00%)
Exposure to solvents or degreasers	4/14 (22.2%)	1/9 (10.0%)	3/13 (18.8%)	0/3 (0.00%)	2/10 (16.7%)
Exposure to welded, brazed or flame cut metal	3/15 (16.7%)	0/10 (0.00%)	3 /14 (17.6%)	0/3 (0.00%)	0/13 (0.00%)
Exposure to metal not otherwise categorized	4/14 (22.2%)	2/8 (20.0%)	2/14 (12.5%)	1/2 (33.3%)	1/12 (7.69%)

[1] Dementia with Lewy Bodies
[2] Multiple System Atrophy
[3] Unspecified parkinsonism corresponds to the heterogeneous parkinsonism cases with not yet defined diagnostic work-up.
[4] Group of rare parkinsonism comprises one case of chronic progressive external ophthalmophegia (CPEO), one case of DYT12 parkinsonism and one case of frontotemporal dementia with parkinsonism (FTD-P).
[5] Vascular parkinsonism

Supplementary Table S5. Clinical characteristics, motor and non-motor symptoms scales in Luxembourg Parkinson's study baseline dataset: diagnostic groups that were not analysed for the reason of small sample size.

	DLB[1] Mean (SD) or YES/NO (%)	MSA[2] Mean (SD) or YES/NO (%)	Unspecified PS[3] Mean (SD) or YES/NO (%)	Rare PS[4] Mean (SD) or YES/NO (%)	Vascular PS[5] Mean (SD) or YES/NO (%)
Individuals per group	19	12	18	3	14
Disease duration since diagnosis (years)	2.32 (2.31)	3.83 (2.86)	2.39 (3.05)	4.33 (2.08)	3.71 (2.79)
Hoehn & Yahr	2.87 (1.15)	3.29 (1.32)	2.14 (0.70)	2.67 (1.15)	2.82 (0.99)
BMI (kg/m2)	26.7 (3.65)	28.4 (4.48)	27.5 (2.56)	23.6 (2.48)	28.1 (4.74)
Obesity	1/15 (6.25%)	4/6 (40.0%)	4/14 (22.2%)	0/3 (0.00%)	5/9 (35.7%)
Metabolic syndrome	1/17 (5.56%)	0/12 (0.00%)	1/17 (5.56%)	0/3 (0.00%)	2/12 (14.3%)
LEDD (gram/day)	0.27 (0.20)	0.63 (0.28)	0.16 (0.18)	0.55 (0.96)	0.33 (0.24)
MDS-UPDRS I	14.9 (5.46)	15.4 (9.01)	11.5 (6.93)	7.33 (4.51)	11.5 (6.50)
MDS-UPDRS II	15.5 (9.64)	25.7 (12.8)	8.00 (5.27)	15.7 (3.06)	12.9 (7.52)
MDS-UPDRS III	46.3 (18.5)	53.2 (22.0)	33.3 (19.8)	37.7 (1.53)	42.1 (18.9)
MDS-UPDRS IV	0.53 (1.61)	2.64 (3.70)	0.00 (0.00)	3.33 (4.93)	0.00 (0.00)
MoCA	16.2 (2.79)	23.2 (4.98)	23.3 (2.97)	25.3 (1.53)	20.4 (5.96)
Sniffin' Stick test	6.88 (3.14)	10.7 (3.34)	10.7 (4.16)	14.3 (1.53)	8.43 (4.38)
Hyposmia	13/4 (76.5%)	5/7 (41.7%)	6/12 (33.3%)	0/3 (0.00%)	8/6 (57.1%)
NMSS	12.1 (5.74)	13.6 (6.50)	8.47 (5.40)	9.67 (6.43)	11.2 (6.18)
SCOPA-AUT	14.1 (5.13)	20.5 (10.2)	14.5 (8.92)	23.0 (7.94)	17.7 (10.6)
RBDSQ	5.56 (4.08)	5.44 (3.97)	4.88 (2.80)	2.00 (1.00)	3.69 (2.72)
probable RBD	6/10 (37.5%)	2/7 (22.2%)	7/10 (41.2%)	0/3 (0.00%)	3/10 (23.1%)
PDSS	98.7 (29.1)	90.1 (30.0)	104 (22.6)	124 (20.7)	96.0 (28.7)
BDI-I	14.4 (7.93)	14.1 (10.0)	9.00 (7.37)	12.3 (0.58)	10.3 (6.09)
PDQ-39	66.5 (35.6)	76.1 (25.2)	33.8 (19.8)	55.7 (16.0)	57.2 (35.6)
Starkstein Apathy scale	14.1 (5.13)	20.5 (10.2)	14.5 (8.92)	23.0 (7.94)	17.7 (10.6)

[1] Dementia with Lewy Bodies
[2] Multiple system atrophy
[3] Unspecified parkinsonism corresponds to the heterogeneous parkinsonism cases with not yet defined diagnostic work-up.
[4] Group of rare parkinsonism comprises one case of chronic progressive external ophthalmophegia (CPEO), one case of DYT12 parkinsonism and one case of frontotemporal dementia with parkinsonism (FTD-P).
[5] Vascular parkinsonism

Supplementary Table S6. Current motor symptoms, non-motor symptoms and comorbidities in Luxembourg Parkinson's study baseline dataset.

	DLB[1] Mean (SD) or YES/NO (%)	MSA[2] Mean (SD) or YES/NO (%)	Unspecified PS[3] Mean (SD) or YES/NO (%)	Rare PS[4] Mean (SD) or YES/NO (%)	Vascular PS[5] Mean (SD) or YES/NO (%)
Individuals per group	19	12	18	3	14
Gait disorder	15/4 (78.9%)	11/1 (91.7%)	9/9 (50.0%)	2/1 (66.7%)	9/5 (64.3%)
Repetitive falls	5/14 (26.3%)	7/5 (58.3%)	4/14 (22.2%)	2/1 (66.7%)	3/11 (21.4%)
Freezing of Gait	4/15 (21.1%)	5/7 (41.7%)	1/17 (5.56%)	1/2 (33.3%)	2/12 (14.3%)
Dyskinesia	1/18 (5.26%)	1/11 (8.33%)	0/18 (0.00%)	1/2 (33.3%)	0/14 (0.00%)
Motor Fluctuations	0/19 (0.00%)	3/9 (25.0%)	0/18 (0.00%)	0/3 (0.00%)	0/14 (0.00%)
Excessive daily sleepiness	8/11 (42.1%)	5/7 (41.7%)	7/11 (38.9%)	0/3 (0.00%)	3/11 (21.4%)
Insomnia	4/15 (21.1%)	6/6 (50.0%)	2/16 (11.1%)	1/2 (33.3%)	2/12 (14.3%)
Orthostatic hypotension	6/13 (31.6%)	7/5 (58.3%)	3/15 (16.7%)	0/3 (0.00%)	3/11 (21.4%)
Syncope	3/16 (15.8%)	4/8 (33.3%)	0/18 (0.00%)	0/3 (0.00%)	0/14 (0.00%)
Dysphagia	3/16 (15.8%)	6/6 (50.0%)	4/14 (22.2%)	2/1 (66.7%)	6/8 (42.9%)
Constipation	9/10 (47.4%)	9/3 (75.0%)	2/16 (11.1%)	1/2 (33.3%)	6/8 (42.9%)
Urinary incontinence	10/9 (52.6%)	9/3 (75.0%)	6/12 (33.3%)	1/2 (33.3%)	3/11 (21.4%)
Hallucinations	9/10 (47.4%)	1/11 (8.33%)	2/16 (11.1%)	0/3 (0.00%)	3/11 (21.4%)
Impulse control disorder	0/19 (0.00%)	1/11 (8.33%)	1/17 (5.56%)	0/3 (0.00%)	2/12 (14.3%)
History or presence of restless legs syndrome	0/19 (0.00%)	1/11 (8.33%)	1/17 (5.56%)	1/2 (33.3%)	0/14 (0.00%)
Diabetes	5/14 (26.3%)	2/10 (16.7%)	4/14 (22.2%)	0/3 (0.00%)	3/11 (21.4%)
Arterial Hypertension	13/6 (68.4%)	6/6 (50.0%)	10/8 (55.6%)	1/2 (33.3%)	11/3 (78.6%)
Cardiovascular disease	6/13 (31.6%)	3/9 (25.0%)	6/12 (33.3%)	1/2 (33.3%)	7/7 (50.0%)
Hypercholesterolemia	9/10 (47.4%)	5/7 (41.7%)	11/7 (61.1%)	1/2 (33.3%)	6/8 (42.9%)
History of stroke	0/19 (0.00%)	1/11 (8.33%)	1/17 (5.56%)	0/3 (0.00%)	9/5 (64.3%)
History of epileptic seizures	1/18 (5.26%)	0/12 (0.00%)	0/18 (0.00%)	0/3 (0.00%)	1/13 (7.14%)
Traumatic brain injury	4/15 (21.1%)	7/5 (58.3%)	3/15 (16.7%)	0/3 (0.00%)	6/8 (42.9%)
History of cancer[6]	2/17 (10.5%)	0/12 (0.00%)	2/16 (11.1%)	0/3 (0.00%)	1/13 (7.14%)
History of melanoma	0/19 (0.00%)	0/12 (0.00%)	0/18 (0.00%)	0/3 (0.00%)	0/14 (0.00%)
History of brain cancer	0/19 (0.00%)	0/12 (0.00%)	1/17 (5.56%)	0/3 (0.00%)	0/14 (0.00%)
History of breast cancer	0/19 (0.00%)	0/12 (0.00%)	1/17 (5.56%)	0/3 (0.00%)	0/14 (0.00%)
History of prostate cancer	2/17 (10.5%)	0/12 (0.00%)	0/18 (0.00%)	0/3 (0.00%)	1/13 (7.14%)
History of cancer not categorized	1/18 (5.26%)	0/12 (0.00%)	0/18 (0.00%)	0/3 (0.00%)	0/14 (0.00%)

[1] Dementia with Lewy Bodies
[2] Multiple System Atrophy
[3] Unspecified parkinsonism corresponds to the heterogeneous parkinsonism cases with not yet defined diagnostic work-up.
[4] Group of rare parkinsonism comprises one case of chronic progressive external ophthalmophegia (CPEO), one case of DYT12 parkinsonism and one case of frontotemporal dementia with parkinsonism (FTD-P).
[5] Vascular parkinsonism
[6] Corresponds to the number of individuals having history of at least one cancer.

Supplementary Table S7. Subanalysis of exposure to the pesticide spraying in the surrounding in people with Parkinson's disease (PD) and controls. Age-relative exposure was calculated as proportion of exposure years to the age at assessment. Pesticides was used as general term standing for any type of pesticides, insecticides, fungicides, herbicides, rodenticides or fumigants. Abbreviations: OR: odds ratio; CI: confidence interval. Significant findings after Bonferroni correction for multiple comparison were annotated by asterisk.

	**Controls** Mean (SD); YES (%)	**PD** Mean (SD); YES (%)	**OR** [*95%* **CI**] (PD vs. controls)
Number of individuals defined (n)	801	685	-
Reported pesticide spraying in the surrounding area	283 (35.3%)	239 (34.9%)	0.98 [0.79;1.21]
Total years of exposure to pesticides in the surrounding are	10.2 (16.3)	10.7 (17.8)	1.00 [1.00;1.01]
Age-relative exposure to pesticide spraying in the surrounding area (in %)	16.3 (25.3)	15.8 (25.6)	1.00 [1.00;1.00]

Supplementary Table S8. Subanalysis of pesticide use at work and at home in people with Parkinson's disease (PD) and controls. Pesticides was used as general term standing for any type of pesticides, insecticides, fungicides, herbicides, rodenticides or fumigants Abbreviations: OR: odds ratio; CI: confidence interval. Significant findings after Bonferroni correction for multiple comparison were annotated by asterisk.

	**Controls** Mean (SD); YES (%)	**PD** Mean (SD); YES (%)	**OR** [**95% CI**] (PD vs. controls)
Number of individuals defined (n)	802	686	-
History of exposure to pesticides (merged occupational or at home use)	547 (68.2%)	417 (60.8%)	0.72 [0.58;0.89]*
Occupational use of pesticides	70 (8.73%)	89 (13.0%)	1.56 [1.12;2.18]*
At-home use of pesticides	533 (66.6%)	403 (58.9%)	0.72 [0.58;0.89]*

Supplementary Table S9. Subanalysis of exposure to the pesticide spraying in the surrounding in people with Parkinson's disease (PD) and controls after exclusion of 1st, 2nd,3rd blood related individuals as well as spouses. Age-relative exposure was calculated as proportion of exposure years to the age at assessment. Pesticides was used as general term standing for any type of pesticides, insecticides, fungicides, herbicides, rodenticides or fumigants Abbreviations: OR: odds ratio; CI: confidence interval. Significant findings after Bonferroni correction for multiple comparison were annotated by asterisk.

	**Controls** Mean (SD); YES (%)	**PD** Mean (SD); YES (%)	**OR** [**95% CI**] (PD vs. controls)
Number of individuals (n)	642	676	-
Reported pesticide spraying in the surrounding area	226 (35.2%)	235 (34.8%)	0.98 [0.78;1.23]
Total years of exposure to pesticides in the surrounding are	9.91 (16.0)	10.7 (17.8)	1.00 [1.00;1.01]
Age-relative exposure to pesticide spraying in the surrounding area (in %)	15.9 (24.8)	15.8 (25.6)	1.00 [1.00;1.00]

Supplementary Table S10. Subanalysis of pesticide use at work and at home in people with Parkinson's disease (PD) and controls after exclusion of 1st, 2nd,3rd blood related individuals as well as spouses. Pesticides was used as general term standing for any type of pesticides, insecticides, fungicides, herbicides, rodenticides or fumigants Abbreviations: OR: odds ratio; CI: confidence interval. Significant findings after Bonferroni correction for multiple comparison were annotated by asterisk.

	**Controls** Mean (SD); YES (%)	**PD** Mean (SD); YES (%)	**OR** [**95% CI**] (PD vs. controls)
Number of individuals defined (n)	643	677	-
History of exposure to pesticides (merged occupational or at home use)	439 (68.3%)	411 (60.7%)	0.72 [0.57;0.90]*
Occupational use of pesticides	54 (8.40%)	87 (12.9%)	1.61 [1.13;2.31]*
At-home use of pesticides	429 (66.9%)	397 (58.8%)	0.71 [0.56;0.88]*

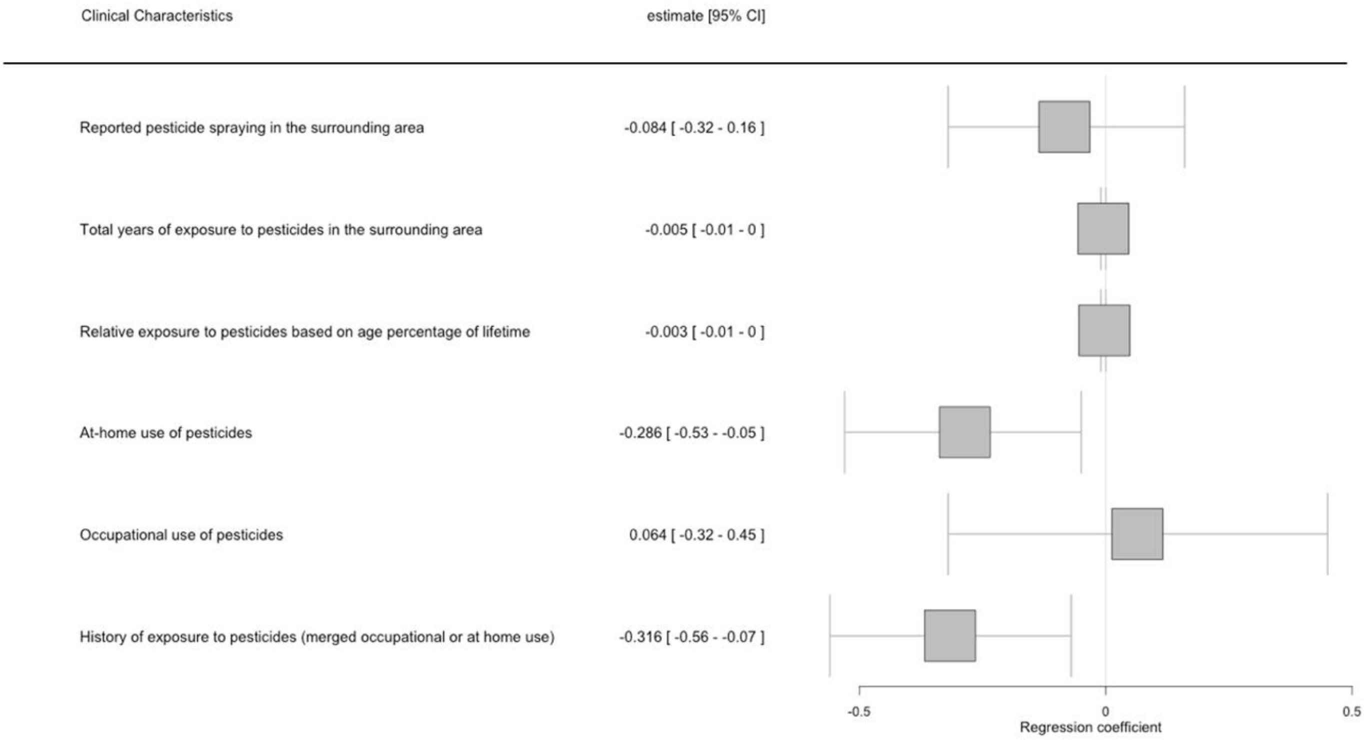

Supplementary Figure S2. Subanalysis of pesticide exposure in people with Parkinson's disease (PD) vs. controls. Results from linear and logistic regression of PD vs. control adjusted for covariates (age at assessment, sex and total languages spoken). The estimates correspond to the regression coefficient with 95 % confidence intervals (95% CI). Significant findings after Bonferroni correction for multiple comparison were annotated by asterisk.

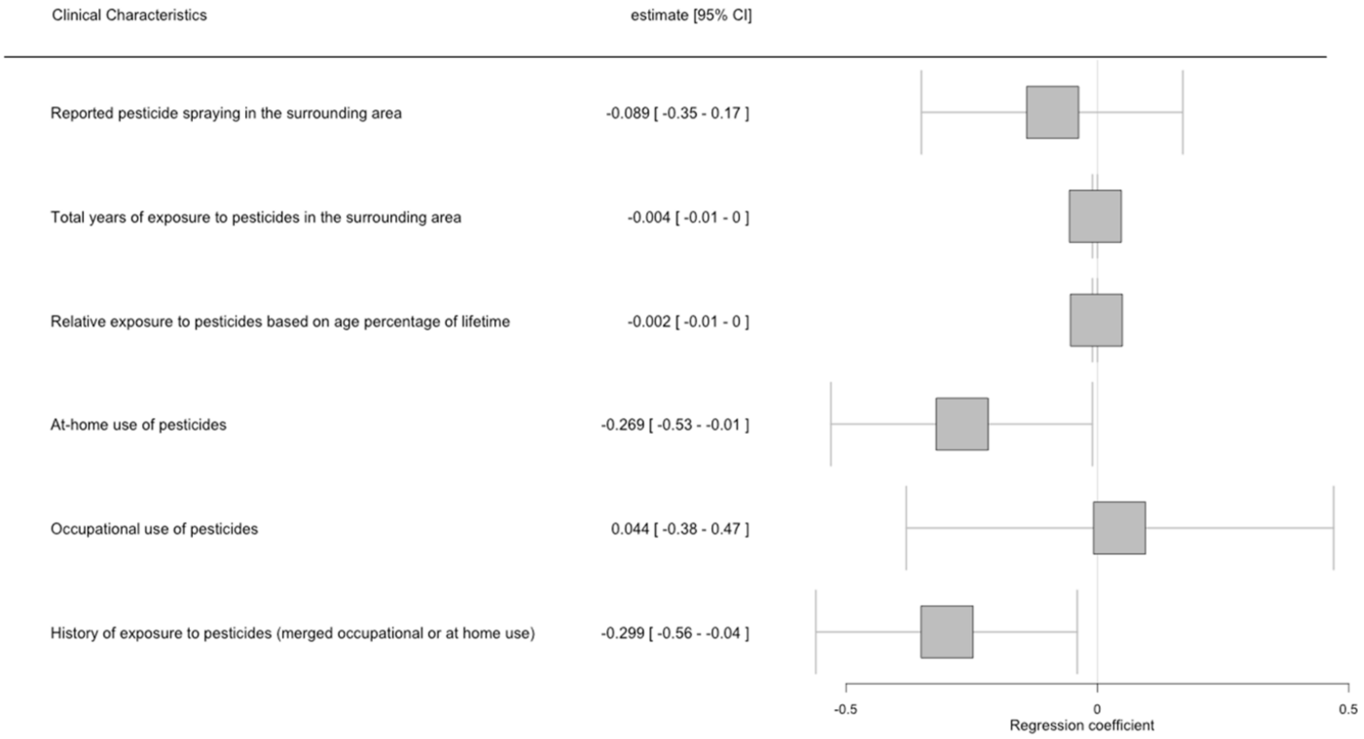

Supplementary Figure S3. Subanalysis of pesticide exposure in people with Parkinson's disease (PD) vs. controls after exclusion of 1st, 2nd, 3rd blood related individuals as well as spouses. Results from linear and logistic regression of PD vs. controls adjusted for covariates (age at assessment, sex and total languages spoken). The estimates correspond to the regression coefficient with 95 % confidence intervals (95% CI). Significant findings after Bonferroni correction for multiple comparison were annotated by asterisk.
